# Recent advances in metal nanoparticles to treat periodontitis

**DOI:** 10.1186/s12951-023-02042-7

**Published:** 2023-08-21

**Authors:** Kamyar Nasiri, Seyed Mohammadreza Masoumi, Sara Amini, Mina Goudarzi, Seyed Mobin Tafreshi, Abbas Bagheri, Saman Yasamineh, Mariem alwan, Meryelem Tania Churampi Arellano, Omid Gholizadeh

**Affiliations:** 1 Department of Dentistry, Islamic Azad University, Tehran, Iran; 2 Faculty of Dentistry, Yazd University of Medical Sciences, Yazd, Iran; 3 School of Science and Engineering, Duquesne University, Pittsburgh, PA USA; 4 School of Dentistry, Dental Research Center, Dentistry Research Institute, Tehran University of Medical Sciences, Tehran, Iran; 5 Department of Endodontics, School of Dentistry, Shahid Sadoughi University of Medical, Yazd, Iran; 6 Research Center for Clinical Virology, Tehran University of Medical Sciences, Tehran, Iran; 7 Medical Technical College, Al-Farahidi University, Baghdad, Iraq; 8 Department of Civil Engineering, Universidad de Lima, Lima, Peru

**Keywords:** Periodontitis, Nanoparticles, Metal nanoparticles, Antibacterial, Antiinflammatory

## Abstract

The gradual deterioration of the supporting periodontal tissues caused by periodontitis, a chronic multifactorial inflammatory disease, is thought to be triggered by the colonization of dysbiotic plaque biofilms in a vulnerable host. One of the most prevalent dental conditions in the world, periodontitis is now the leading factor in adult tooth loss. When periodontitis does develop, it is treated by scraping the mineralized deposits and dental biofilm off the tooth surfaces. Numerous studies have shown that non-surgical treatment significantly improves clinical and microbiological indices in individuals with periodontitis. Although periodontal parameters have significantly improved, certain bacterial reservoirs often persist on root surfaces even after standard periodontal therapy. Periodontitis has been treated with local or systemic antibiotics as well as scaling and root planning. Since there aren't many brand-new antibiotics on the market, several researchers are currently concentrating on creating alternate methods of combating periodontal germs. There is a delay in a study on the subject of nanoparticle (NP) toxicity, which is especially concerned with mechanisms of action, while the area of nanomedicine develops. The most promising of them are metal NPs since they have potent antibacterial action. Metal NPs may be employed as efficient growth inhibitors in a variety of bacteria, making them useful for the treatment of periodontitis. In this way, the new metal NPs contributed significantly to the development of efficient anti-inflammatory and antibacterial platforms for the treatment of periodontitis. The current therapeutic effects of several metallic NPs on periodontitis are summarized in this study. This data might be used to develop NP-based therapeutic alternatives for the treatment of periodontal infections.

**Graphical Abstract:**

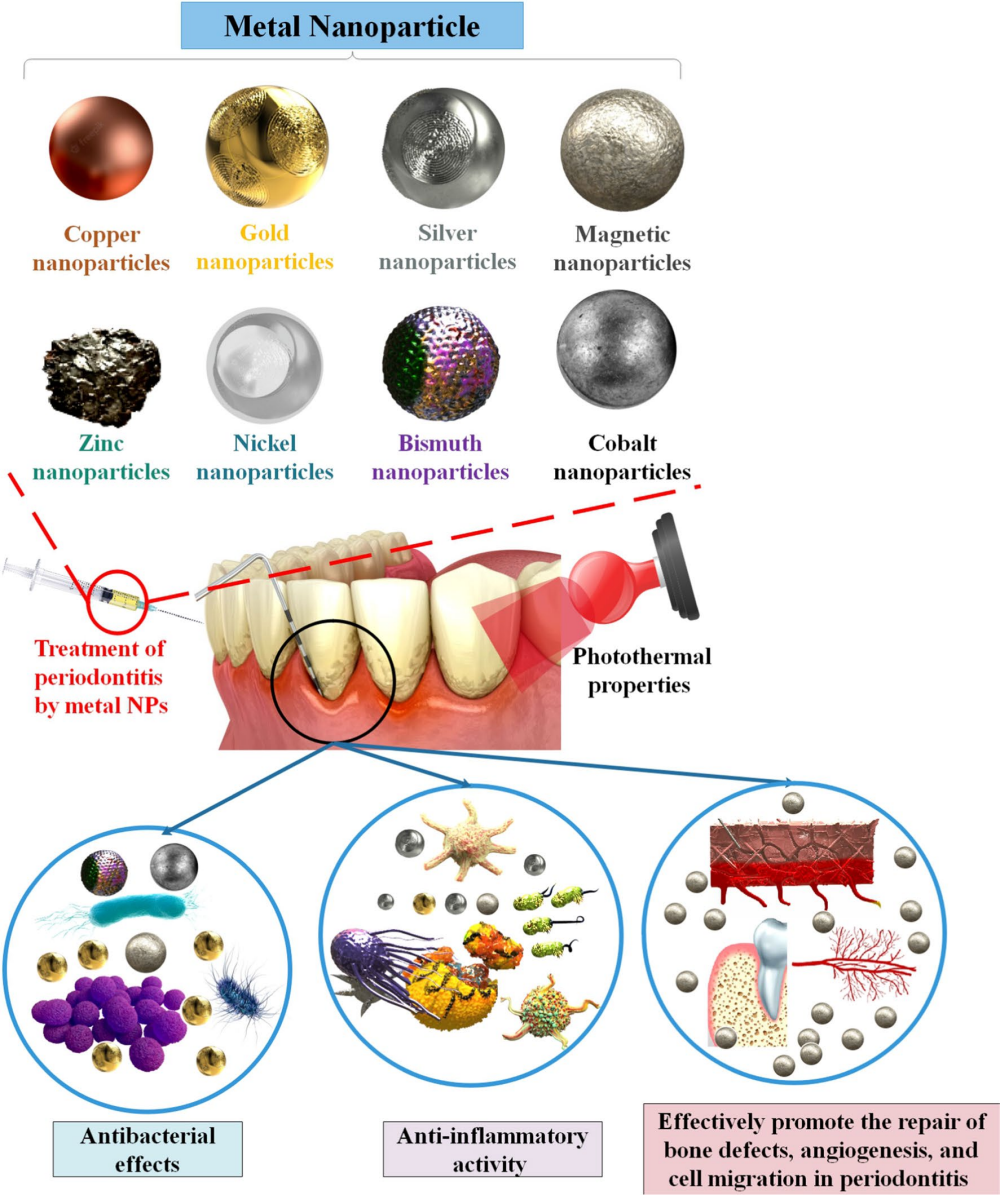

## Introduction

Before the turn of the century, people often used the word “pyorrhea” to describe periodontal disease, which is characterized by pus, periodontal pockets, bone loss, and tooth loss. It was generally accepted that pyorrhea and the subsequent tooth loss were natural consequences of becoming older. Periodontal disease has become more important as a root cause of tooth loss in recent years, especially while the prevalence of dental caries has decreased in the general population. As the number of teeth lost to caries declines, the number of teeth at risk of periodontal disease rises [1]. Deterioration of periodontal ligaments (PDLs), the development of periodontal pockets, and the resorption of alveolar bone are hallmarks of periodontitis, an inflammatory disease of the gums that destroys the foundation on which teeth rest. Pockets may get infected with periodontitis because of the proliferation of microflora, especially anaerobes, which produce toxins, enzymes, and immune system stimulation [2]. This is often linked to a variety of clinical diseases, including gum inflammation (gingivitis), PDL and dental cementum deterioration, and loss of alveolar bone. The disease's early stage, gingivitis, may evolve into periodontitis, a more serious illness that manifests as the growth of cavities, a loss of clinical connection, and the deterioration of alveolar bone [3]. The use of pharmacological medicines, mechanical therapy, and surgical intervention are only a few of the several methods used to treat illness. Antimicrobials that alter the microbial flora in the periodontal milieu and host response modulating agents that alter host responses like lowering excessive enzyme levels, cytokines, prostaglandins, and osteoclast activity are among the medications specifically used for improving the management of periodontitis [4]. Osteoclasts, osteoblasts, and bone marrow stromal cells were traditionally considered to regulate bone loss in periodontitis. In addition to starting the physiological bone remodeling, osteocytes have recently been discovered to help in inflammation-related bone remodeling [5]. Evidence from recent case–control and cross-sectional studies suggests that periodontitis may raise the chance of having a prematurely born baby with a low birth weight by a factor of seven, and the risk of cardiovascular disease by a factor of two. These preliminary studies suggest a possible connection between general and oral health. Evidence from this research also lends credence to the idea that periodontal disease is the result of an inflammatory response from the host on both the local and systemic levels [6]. Without treatment, periodontitis, an inflammatory condition caused by bacteria, destroys the tissues around your teeth and eventually forces you to lose them. An imbalance between the host's immunological protection and immune destruction processes is a major contributor to periodontal tissue deterioration. To restore the physiological structure and function of the periodontium, periodontal treatment seeks to reduce inflammation, and repair and regenerate hard and soft tissues. Thanks to recent developments in nanotechnology, immunomodulatory nanoparticles (NPs) may now be used in regenerative dental procedures [7].

NPs can efficiently enter biological organisms due to their very tiny size. The effective and targeted delivery of medications, genes, and therapeutic chemicals to specific organs or cells, imaging, and precise identification of viruses at early stages are only a few examples of the many applications of nano biomedical expertise. NPs also play a crucial role in antiviral therapy by improving the efficacy of medicine use and facilitating the transfer of hydrophobic drugs [8–10]. Due to their small size (1–100 nm), NPs can easily pass through even the smallest blood capillaries and avoid being phagocytized, which increases their plasma half-life and allows for a more gradual release of the drug. The NPs may be classified as either “organic” or “inorganic”. Polymers, dendrimers, micelles, liposomes, and lipids are all examples of organic materials; silver (Ag), gold (Au), iron oxide, zinc oxide (ZnO), and silica are all examples of inorganic materials [9]. NPs have several uses, including medication delivery at a precise point, sensors, pharmaceuticals to combat cancer, antioxidants, larvicides, Nanofluids, antibacterial agents in dentistry, mouthwashes, the treatment of white spot lesions during orthodontic therapy, and more. Since the size reduction limits the electron movements, NPs have superior optical characteristics compared to bulk metals. They are less hazardous and have a high surface-to-volume ratio because of their very tiny size. Therefore, quicker absorption and a relatively greater drug loading arise from interactions at the surface. NPs' increased antibacterial action may be attributed to their huge surface area and high charge density, which allows them to interact with the negatively charged surface of bacterial cells [11]. Nanotechnology has a wide variety of potential dental applications. Nano-products, regenerative dentistry, periodontics, implantology, periodontal therapy, endodontics, prosthodontics, conservative and cosmetic dentistry, orthodontics, and endodontics are all part of the dental care industry. Dental equipment, such as LED light curing units, dental hard tissue protection against acid-containing meals, dental material characterization, and dental hard tissue characterization, all rely heavily on nanotechnology [12]. Biomineralization is sparked by metallic NPs, which promote the remineralization of demineralized (caries-ridden) dental tissues. In addition, because of their ion balance in oral fluid, metallic NP may triumph over obstacles under a wide variety of oral circumstances. Many different nano-formulations have been studied by academics and clinicians for their ability to reduce caries [13]. In addition, there's evidence that metal NPs may inhibit the growth of harmful germs. Numerous studies have been conducted so far on the efficacy of various metal NPs against pathogenic microorganisms [14]. Metal NPs, according to several studies, are an appealing new approach to treating periodontitis because of their ability to enhance the photothermal characteristics and anti-inflammatory action of materials [15]. Metal NPs, which have antibacterial and anti-inflammatory characteristics, are also being considered as potential periodontitis treatments. For instance, a dose- and time-dependent increase in cytotoxicity was seen in human periodontal fibroblasts exposed to silver NPs (AgNPs) smaller than 20 nm [16, 17]. In this paper, we discussed metal NPs, which are useful in the treatment of periodontitis.

## Development factors of periodontitis

It was predicted that by 2020, almost 62% of dentate individuals will have periodontitis, with a further 23.6% suffering from severe periodontitis. When compared to estimates provided between 1990 and 2010, these findings reveal an alarmingly high frequency of periodontitis [18]. Inflammation of the gums and loss of alveolar bone that supports teeth characterize periodontitis, the most prevalent oral illness. Plaque biofilm on the dental and gingival surfaces is the primary cause of periodontal disease, which in turn is governed by unclean behavioral factors, the internal milieu of the oral cavity, and the production of dental and gingival plaque. Inducing host immune responses that harm gingival tissues and resorb bone, *Porphyromonas gingivalis* (*P. gingivalis*) is the main keystone pathogen of the periodontal biofilm [19]. Demineralization and destruction of tooth hard tissues characterize dental caries, also known as tooth decay, which may advance to inflame and destroy the soft tissues around the teeth, leading to periodontitis. There are several potential causes of dental caries and periodontal disease. The oral microbiome has been examined extensively, both in healthy people and those with diseases. The oral cavity is home to bacteria from at least 13 distinct phyla: *Actinobacteria*, *Bacteroidetes*, *Chloroflexi*, *Firmicutes*, *Fusobacteria*, *Proteobacteria*, *Spirochaetes*, *Synergistetes*, *Tenericutes*, and the as-yet-unnamed *SR1* and *TM7* [20]. It indicates that the number of spirochetes present in subgingival plaque is proportional to the clinical stage of periodontal disease. The exact number of spirochetal species that colonize the plaque is unknown, but we can tell that there are three sizes of spirochetes: tiny, medium, and giant. Among the four cultivable species of tiny spirochetes, *Treponema denticola* (*T. denticola*) is the only one that has been proven to contain proteolytic and keratinolytic enzymes, as well as factors or processes that reduce lymphocyte blastogenesis and inhibit fibroblast and polymorphonuclear leukocyte (PMNL) activity. Periodontal tissue injury may result from any or all of these factors [21]. An individual’s reaction to periodontal infection is significantly influenced by risk factors. Lifestyle variables, such as smoking and alcohol intake, are examples of these separate but changeable risk factors for periodontal disease. Conditions including diabetes mellitus, obesity, metabolic syndrome, osteoporosis, and inadequate calcium and vitamin D intake are also included. The modern treatment of many periodontal patients includes the control of these modifiable risk factors. Because of the role played by genetic variables, it is possible to target people for periodontal disease prevention and early diagnosis. Aggressive periodontitis has a definite hereditary component. Despite widespread speculation that some genes may play a role in the development of chronic adult periodontitis, no definitive evidence linking these two conditions has been found [22].

## Periodontitis treatment methods

In most cases, opportunistic infections are the root cause of periodontal disease. Due to the infection caused by bacteria in a biofilm that is resistant to antimicrobials and the body’s natural defenses, periodontal disease is difficult to treat. It is a time-consuming process to eliminate the germs from the periodontal cavity, and the bacteria persist after treatment. The germs present and the host's reaction both play major roles in determining the severity of the illness [23]. Gingival swelling, bleeding gums, and bad breath are all symptoms of periodontitis, which begins as a gingival (gingivitis) inflammation and progresses into deeper tissues. Pockets arise when alveolar bone resorbs, gingival epithelial tissue migrates, and degenerating collagen in the periodontium provides less support for the teeth. Therefore, the progression of the condition determines the therapeutic approach that will be used [24]. Teeth might become loose and fall out if this condition is not corrected. Inflammation and degeneration of the gums, PDL, and cementum (the substance that holds teeth in place) are hallmarks of this condition. Periodontal pockets are created when gingival epithelium migrates along the tooth surface. These pockets are a fertile breeding ground for bacteria. The teeth get loose and eventually fall out in the latter stages. Local delivery offers the benefit of producing larger medication concentrations at the desired site of action while requiring lower dosages, which results in a decrease in harmful and side effects. For instance, syringes, irrigation tools, dental gels, mouthwashes, and gentrifiers. Due to the drug's brief interaction with tissues and inadequate penetration into the periodontal pocket, mouth rinses, and dental treatments are regarded as ineffective. Dental irrigation aids in the decrease of dental plaque and subgingival bacteria [25]. The primary objective of periodontitis treatment is to halt the progression of the disease, thereby decreasing the likelihood of tooth loss, alleviating symptoms and the perception of the disease, potentially restoring lost periodontal tissue, and offering guidance on maintaining a healthy periodontium. Therapeutic intervention encompasses the implementation of strategies aimed at modifying behavior, including personalized oral hygiene instructions, smoking cessation programs, dietary modifications, subgingival instrumentation for plaque and calculus removal, local and systemic pharmacotherapy, as well as diverse surgical procedures. There is no definitive evidence to support the superiority of any specific treatment option, and nearly all forms of mechanical periodontal treatment experience positive effects when combined with adjunctive antimicrobial chemotherapy [26]. By stopping the chronic inflammatory process that leads to the loss of periodontal attachment, alveolar bone, and the development of periodontal pockets, periodontal treatment aims to retain the natural dentition. Periodontitis' etiology and pathogenesis are now understood to be the consequence of a complex interaction between bacterial aggressiveness and host defense, which is influenced by behavioral and systemic risk factors. Since only treatments that mechanically disrupt subgingival biofilms are effective, maintaining periodontal health requires that patients adequately manage their plaque and that professionals do periodic professional prophylaxis [27]. Fluorides are often utilized, and their antiseptic qualities help lessen gingivitis and bacterial buildup. Antibiotic usage may lessen periodontal disease occurrence and severity [28]. Chlorhexidine (CHX) gluconate is a frequently employed antimicrobial compound that is utilized in conjunction with mechanical periodontal therapy. Typically, it is administered in the form of an oral rinse; however, alternative applications include gel, varnish, and subgingival chip formulations. The utilization of CHX, in conjunction with routine tooth brushing, has the potential to result in a decrease in the accumulation of dental plaque, thereby offering significant advantages in the management of chronic periodontitis. Pharmacotherapy for periodontal disease has witnessed a recent emergence as a notable advancement. The CHX gluconate chip is introduced into the periodontal pocket after the completion of the cleaning procedure, facilitating a protracted and controlled discharge of CHX gluconate within the targeted region. Systemic antibiotics are occasionally necessary, however, they are uncommon, as in the case of chronic deep periodontal pockets. Tetracyclines, penicillin, macrolides, quinolones, cephalosporins, and nitroimidazole compounds are the most often prescribed antimicrobial drugs. These pharmaceutical treatments can be provided to patients with a variety of susceptible germs, including some that are resistant to antibiotics, and they all have different mechanisms of action. These medications can also be administered separately or in combination to increase their potential uses [29, 30]. Following invasive periodontal operations, patients are frequently prescribed antibiotics as a preventative measure. Antibiotic resistance has, however, recently grown in patients with periodontal disease, in line with the general trend of rising antimicrobial resistance in human pathogens. These bacteria are less vulnerable to antibiotics because of the particular periodontal environment and biofilm production. To treat periodontal disorders, new treatment approaches are required [31, 32]. In addition to controlling supragingival plaque, manual, sonic, and/or ultrasonic instrumentation is another non-surgical periodontal therapy option. It was discovered that subgingival debridement was a successful therapy for lowering the depth of the probing pocket and raising the clinical attachment level. Treatment reduces the depth of the pocket because the gingiva recedes and the clinical attachment level rises. The therapy is deemed effective when a pocket depth of less than or equal to 5mm is attained. Periodontal treatment is negatively impacted by patient-related variables like smoking status and the severity of the illness. Endodontic therapy and tooth type are two site-specific variables that may affect the result. There is a distinction between single-rooted and multi-rooted teeth with the potential for furcation involvement about tooth type, which is a challenge for the effective treatment of molars [33]. In addition, the removal of the periodontal pocket and the clearing of the subgingival infection are regarded as essential in the treatment of periodontitis. It seems that periodontal disease can be treated well in as little as one to three days, albeit this does not prevent the illness from being recolonized and returning. It's possible that utilizing many modes of administration simultaneously yields the best results. The utilization of a short-acting biodegradable system as an initial treatment may prove advantageous in delivering a bactericidal concentration of the antibacterial agent directly to the periodontal pocket. The continued and extended administration of antibacterial agents to the vicinity surrounding the opening of the pocket can potentially hinder the re-establishment of bacteria in the pocket from the oral cavity by suppressing the formation of plaque at the margins [27, 34].

**Fig. 1:**
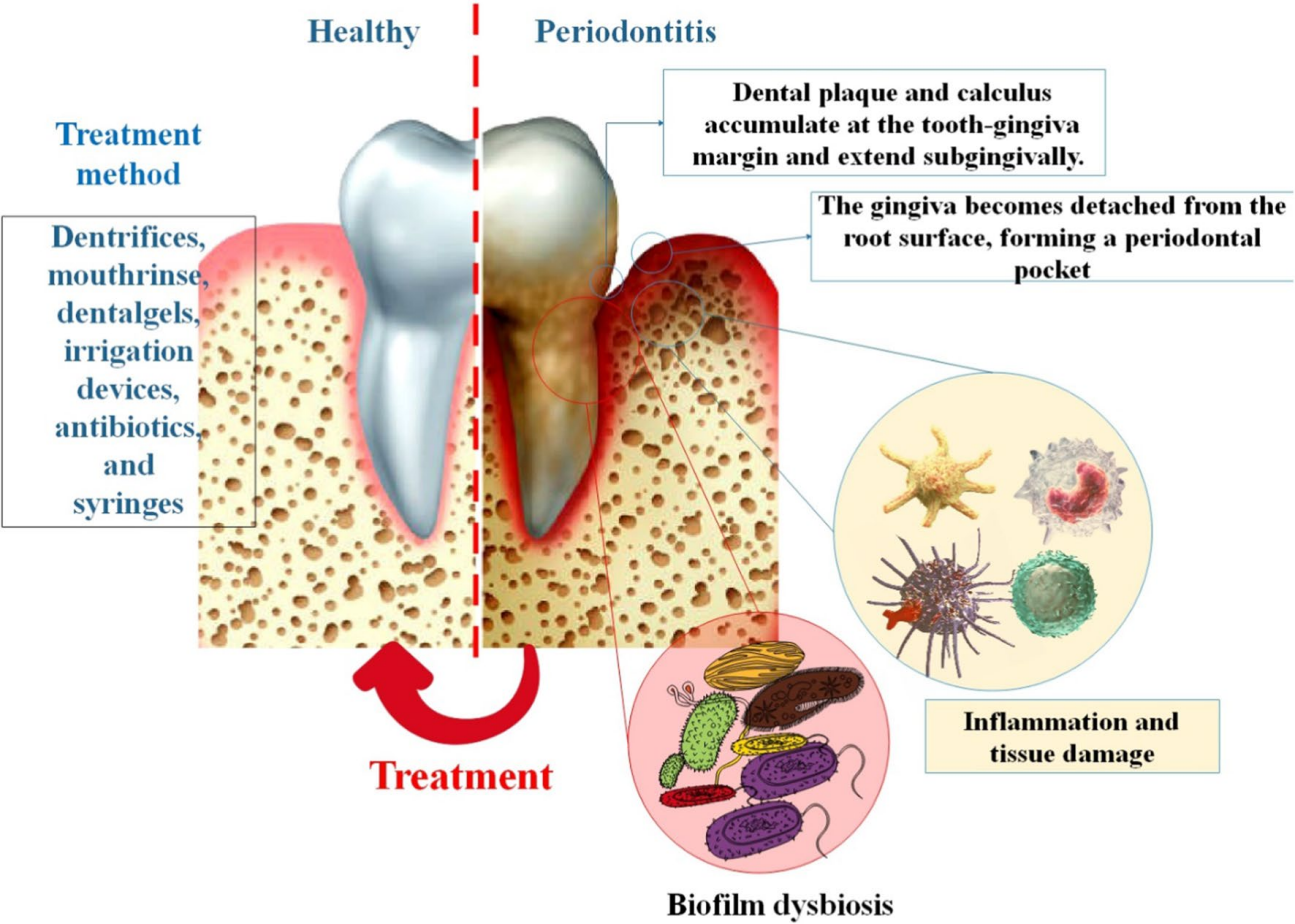
Factors that stimulate periodontal disease, such as biofilm dysbiosis, uncontrolled gingival and periodontal inflammatory reactions, psychological stress paralleled by increased cortisol discharge, and harmful foods defined by high carbohydrate consumption. Dental plaque and calculus accumulate at the tooth-gingiva margin and extend subgingivally. The activities of subgingival plaque and host protection result in inflammation and tissue injury. The gingiva becomes detached from the root surface, creating a periodontal pocket, which is extremely anaerobic and allows additional expansion and development of subgingival plaque. Increasingly severe destruction of tissues results in the gradual recession of the supporting alveolar bone [41, 42]

Periodontitis would be brought on by the oral pathogenic bacteria' biofilm, which is brought on by their coaggregation and growth. Individual bacteria are encased in biofilms by extracellular polymeric substances (EPS), acting as a barrier to keep them safe from harm. Disrupting the EPS of pathogenic bacteria is essential and problematic for the treatment of periodontal disease. Researchers assumed that our specially developed cationic dextrans may be effective in treating periodontitis based on their ability to sufficiently disorganize EPS. Researchers confirmed that cationic dextrans may cause EPS biofilms, particularly those containing *P. gingivalis*, a major periodontal pathogen, to undergo a phase shift, thereby eliminating the biofilm in vitro. More significantly, a rat model of periodontal disease had a favorable in vivo therapy. In conclusion, the research took use of the substantial biofilm-controlling ability of cationic dextrans to treat periodontitis practically and successfully [35]. Despite being the Au standard for treating periodontal disease, non-surgical periodontal therapy (NSPT) might nevertheless provide subpar outcomes because of anatomical and microbiological restrictions. Probiotic usage as a supplement to non-surgical periodontal therapy may be able to enhance periodontal clinical parameters for up to three months, according to limited data [36]. Nevertheless, the current dental materials, instruments, and procedures exhibit limitations in terms of their efficacy and precision in targeting microbial pathogens, particularly in reaching deep periodontal pockets. Nano-drug delivery systems present a sophisticated approach to drug delivery in the context of periodontitis, exhibiting efficacy against pathogens that have developed resistance. NPs have been widely utilized in diverse dental applications owing to the distinctive characteristics that render them appropriate for drug delivery purposes. The small dimensions of NPs enable targeted drug delivery to specific tissues, cells, or pathogens within the periodontal pockets. Furthermore, they exhibit antimicrobial properties through the disruption of bacterial cell membranes, resulting in the eradication of bacteria. The utilization of inorganic NPs offers several benefits, including their notable surface-to-volume ratios, diverse shapes, numerous structural defects, such as irregularities in the crystal lattice of nanostructured bismuth oxide, and their nanoscale dimensions. These attributes enable a greater number of active sites for interaction with various biological systems, such as bacteria, fungi, and viruses. The primary distinction between NPs and conventional organic molecular antimicrobial agents lies in their significance, as it has the potential to mitigate the emergence of antimicrobial resistance [37–40]. In Fig. 1, we have shown the provoking factors of periodontal disease and the methods of non-surgical treatment of periodontitis.

## Metal nanoparticles in periodontitis

**Fig. 2:**
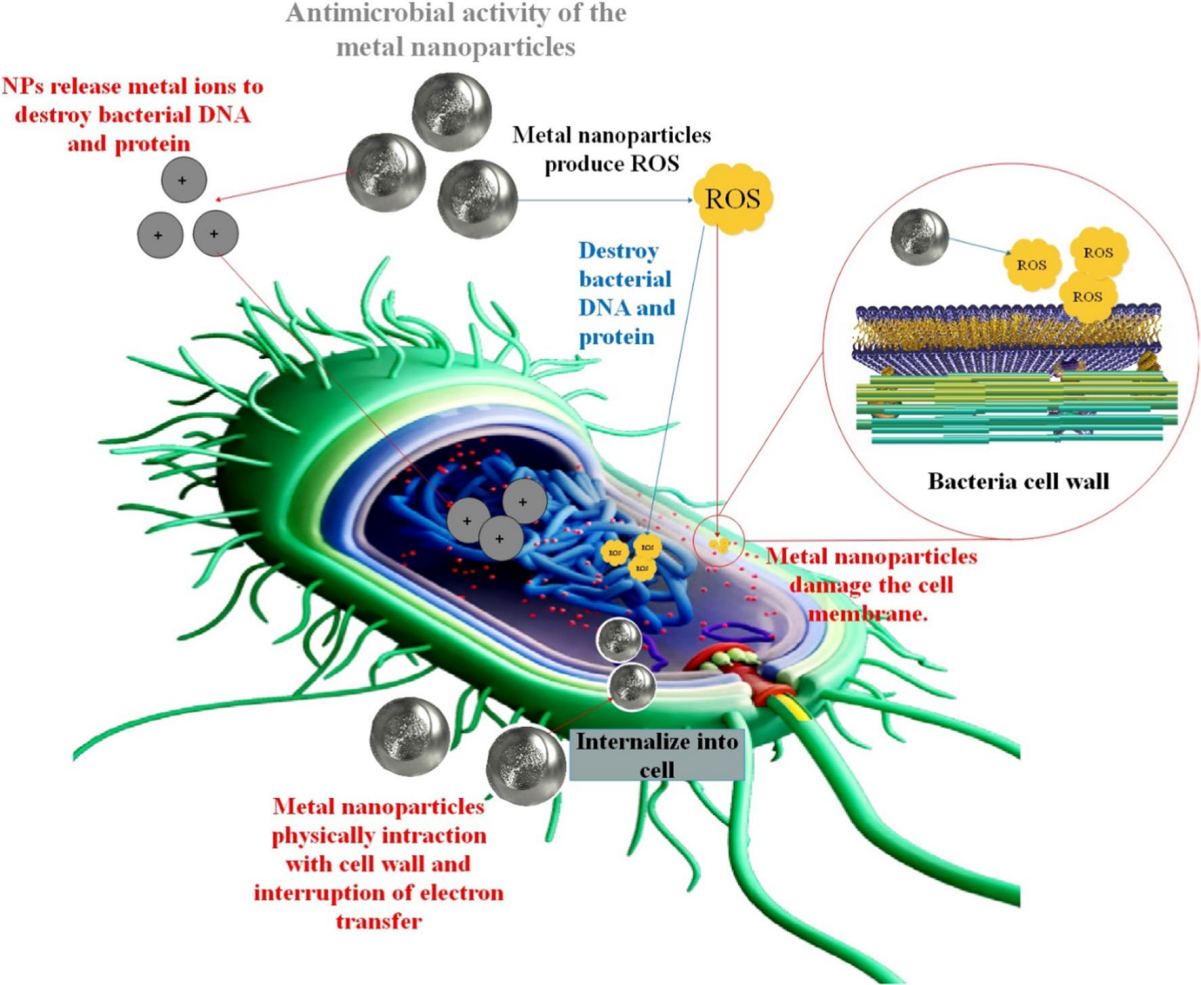
Various mechanisms of antimicrobial activity of the metal NPs. Native bacteria without metal NPs. **c** metal NPs destruct DNA.

Generally, smaller NPs tend to be more effective in killing germs. However, bigger NPs have been proven to be more effective in other trials, suggesting that size is not the primary determinant of toxicity. The formulation procedure, the surrounding conditions, the bacterial resistance mechanism, and the NP’s physical properties are all additional variables [43]. The antibacterial effect of distinct transient metal and metal oxide NPs has been shown against a wide range of Gram-negative and Gram-positive bacteria, including pathogens and those resistant to standard antibacterial drugs. Therefore, NPs may be used in nanotechnology to inhibit bacterial development and in biomedicine to remedy a wide range of illnesses [44]. There has been a lot of interest in the possible antibacterial properties of various NPs and their derivatives. Antimicrobial activity in metal NPs was discovered, including gold Au, Ag, Ag oxide (Ag2O), titanium dioxide (TiO2), silicon (Si), copper oxide (CuO), ZnO, Au, calcium oxide (CaO), and magnesium oxide (MgO) [45]. The reactive oxygen species (ROS) generated by these NPs are toxic to microbes because they may degrade DNA, RNA, and proteins. Since their antibacterial action does not rely on ROS, it seems that Au NPs are less toxic to mammalian cells than the other nanometals. Additionally, the great functionalization potential of these NPs makes them suitable nanomaterials for use as targeted antibacterial agents. In addition, the antibacterial efficacy of ZnONPs is enhanced by their excellent photocatalytic activities. ROS are also produced by ZnONPs when exposed to UV radiation [46]. To do their part, metal NPs eliminate the biofilm. An essential contributor to the resurgence of drug-resistant bacteria that has resulted in a dramatic rise in mortality and morbidity and a drawn-out treatment cycle is the development of biofilms [47, 48]. Furthermore, the metal ions released by these NPs kill bacterial DNA and protein. In the case of metal NPs, the positive metal ions interact with the negative membrane charges of microorganisms. Metal ions can cross the membrane of bacteria and enter the cells. They prevent protein and nucleic acid production by reacting with the sulfhydryl group (–SH) on microbial proteins. NPs’ antibacterial efficacy is dependent on their size and surface charge. Excellent antibacterial effects are achieved with no degradation of the material's mechanical qualities because of their reduced particle size and higher surface-to-volume ratio. The release of the loaded antibacterial chemical is another way for killing bacteria, in addition to the one described above that involves direct contact with NPs [48]. The antibacterial mechanisms of metal NPs are summarized in Fig. 2. In addition to being a leading cause of adult tooth loss, periodontitis has been linked to the chronic activation of the host immune system and has been shown to have bidirectional effects with various systemic disorders such as diabetes, cardiovascular disease, digestive disease, and cancer. Therefore, protecting and restoring periodontal health is crucial to overall wellness. Therefore, it is crucial to find a way to treat periodontitis that is both more successful and less intrusive [49]. Due to their low toxicity, photothermal capabilities, immunotherapy, anti-inflammatory activity, antibacterial qualities, simple manufacturing techniques, and cheap cost, multifunctional metal NPs with therapeutic activities show considerable promise in the domains of periodontal disease treatment [50, 51]. In addition, several potential regenerative drugs, including those based on polymeric and inorganic NPs, have recently been developed thanks to advances in nanotechnology. One of the most effective NPs is the mNP, which has undergone significant research for biological purposes such as promoting cell mineralization and osteogenic differentiation. For instance, AuNPs’ impact on the development of osteogenic stem cells has been shown. Researchers also demonstrated how the size of AuNPs affected how much the human PDL progenitor cells differentiated into osteogenic cells. The use of AuNPs still requires improved design and targeted targeting of the molecular pathways to regulate cell differentiation despite the enormous work in this sector to date [52] (Table 1).

**Table 1:** Metal NPs are often used as an antibacterial agent in periodontitis

Nanoparticles	Bacterial infection	Study type	Antibacterial effects	Refs.
AuNPs	*Staphylococcus aureus* (*S. aureus*), *Escherichia coli* (*E. coli*), and* S. aureus*	In vivo and in vitro	Dental plaque biofilm was prevented by E-Au@H with near-infrared (NIR) light irradiation by 87%, and alveolar bone repair was aided by 38%	[53]
AgNPs	*Streptococcus mutans (S. mutans)*, *S. aureus, Lactobacillus*, and *Candida albicans (C. albicans)*	In vitro	At a concentration of 100 L/mL, the AgNPs-based mouthwash showed antibacterial efficacy against *Lactobacillus*, *C. albicans*, *S. mutans*, and *S. aureus*	[54]
AgNPs	*P. gingivalis*	In vitro	When used in conjunction with other antimicrobial agents, AgNPs may have a synergistic impact and be employed for local drug administration during periodontal treatment as an alternative to topical antiseptics and antibacterial agents	[55]
AuNPs	*P. gingivalis*	In vivo and in vitro	The coated aligners showed favorable antibacterial activity against *P. gingivalis*. This study reveals a new method for treating oral *P. gingivalis* by coating aligners with 4,6-diamino-2-pyrimidinethiol-modified AuNPs (AuDAPT), which has typical advantages compared to other treatments for both periodontitis and related systematic diseases	[56]
Ag/Au NPs	*P. gingivalis*	In vitro	Ag/Au NPs inhibited the planktonic growth of *P. gingivalis* W83. This effect was enhanced in the presence of hydrogen peroxide, which simulates the oxidative stress environment in the periodontal pocket during chronic inflammation	[57]
ZNONPs	*S. mutans* and *P. gingivalis*	In vitro	In comparison to the plain PCL membranes, *S. mutans,* and *P. gingivalis* adherence was dramatically reduced in the LZ and HZ groups that included ZnO	[58]
TiO2 nanotubes	*A. actinomycetemcomitans*, *T. forsythia*, and* Campylobacter*	In vitro	Ag-doped TiO2 nanotubes that had not yet been annealed showed a strong Ag peak. Bacterial mortality against *A. actinomycetemcomitans*, *T. forsythia*, and *C. rectus* was seen against the as-annealed Ag-doped TiO2 nanotubes, demonstrating antibacterial effectiveness	[59]
Fe_3_O_4_NPs	*S. sanguinis*, *P.* gingivalis, and *F. nucleatum*	In vitro	By using a magnetic field to destroy bacteria, Fe3O4 with a magnetic field permitted the targeting of infection locations	[60]
NiNPs	*S. aureus*, *S. epidermidis* and *E. coli*	In vitro	The goal of the current investigation was to ascertain if NiNPs had any inhibitory effects on the biofilm formation of clinical isolates of *S. epidermidis*. *S. epidermidis* has a primary virulence factor that includes the capacity to form biofilms on viable and non-viable surfaces, particularly on plastic devices	[61]
BiNPs	S. *mutans*	In vitro	The introduction of zero-valent BiNPs completely stopped *S. mutans* from producing biofilm. This result was surprising since zero-valent BiNPs were anticipated to have an inhibitory effect on cell growth but not a complete block. Researchers hypothesized that because 69% of the cells were rendered inactive by NPs, there wouldn't be enough cells left to form a biofilm	[62]
BiNPs	*E. faecalis* and* S. mutans*	In vitro	Given the benefits of BiNPs, such as their ability to suppress biofilm formation and their increased antibacterial activity compared to CHX, they may be a promising alternative to CHX in the fight against *E. faecalis* that might be proposed for use in several branches of dentistry	[63]
CoNPs	S*. aureus* and *E. coli*	In vitro	CoNPs were observed from 0.125 to 128.0 µg/ml against S. aureus and *E. coli.* The zone of inhibition of CoNPs was better against *E. coli* than *S. aureus*	[64]
CuNPs	*A. Actinomycetemcomitans*	In vitro	The 100 g/mL copper-content solid sponges and gel spheres were made using CuNPs /chitosan gel nanocomposites. The development of *A. Actinomycetemcomitans* were stymied by these substances. The sphere nanocomposites were more stable in saliva and showed a prolonged release of copper at amounts effective against bacteria	[65]
CuNPs	*S. mutans*	In vivo and in vitro	The composite ceramic's copper ions damage the permeability of the *S. mutans* membrane, which in turn disrupts the bacterial respiratory system and DNA replication, ultimately leading to the death of the organism	[66]

### Properties of gold nanoparticles

A drug carrier with excellent bioavailability and functionality, gold NPs (AuNPs) have a diameter between 50 and 150 nm. Photothermal treatment, which uses a specific wavelength of light exposure and the mechanical capabilities of AuNPs as a photo-absorbing agent to promote light conversion into heat leading to protein degradation, is often used to treat dental problems [67]. AuNPs have also been shown to be a fantastic option for use as an antibacterial agent. Analysis of AuNP's antibacterial activity has also been a hot topic in modern times. AuNP has been shown to have an acceptable performance against a variety of infections [68]. Dental caries, gum disease, tissue engineering, dental implantology, and cancer diagnostics have all been studied using AuNPs. Since AuNPs have antifungal and antibacterial properties, they are added to other biomaterials to boost their effects. They also increase the mechanical qualities of materials, producing better results. They come in a variety of sizes and concentrations to demonstrate their therapeutic effects. These characteristics of AuNPs make them a popular filler for biomaterials [69].

#### Gold nanoparticles in periodontitis

In a study, it was found that 45 nm AuNPs could significantly reduce inflammation and improve the periodontal inflammatory microenvironment by controlling the production of pro-inflammatory and anti-inflammatory cytokines and controlling macrophage polarisation, which in turn affected the differentiation of human PDL cells (hPDLCs). The interaction between AuNPs-conditioned macrophage and AuNPs-stimulated hPDLCs greatly increased the periodontal tissue differentiation capability of hPDLCs in the LPS-activated inflammatory macrophage-hPDLCs coculture system, in addition to the direct effects of AuNPs on hPDLCs. Using 45nm AuNPs has been shown to considerably slow the progression of periodontitis and increase the amount of new periodontal attachment, bone, and cementum formed in periodontal defects. In addition to influencing the early inflammatory response of periodontal tissues by modulating macrophage phenotypes, investigation demonstrated that 45 nm AuNPs may directly affect hPDLCs. This resulted in a milieu with limited amounts of inflammatory cytokines and reparative cytokines including bone morphogenetic protein-2 (BMP-2), which stimulated PDLC development, periodontal tissue regeneration, and the arrest of periodontitis progression [70]. Another look into how chiral-modified AuNPs affect osteogenic differentiation, hPDLC autophagy, and periodontal tissue regeneration was conducted. L/D-cysteine-anchored AuNPs (L/D-Cys-AuNPs) were successfully synthesized in this work. L-Cys-AuNPs are better absorbed by hPDLCs than D-Cys-AuNPs in vitro. Furthermore, L-Cys-AuNPs significantly decreased the expression of sequestosome 1 and significantly increased the expression of alkaline phosphatase (ALP), collagen type 1, osteocalcin, runt-related transcription factor 2, and microtubule-associated protein light chain 3 II in hPDLCs compared to expression levels in hPDLCs treated with D-Cys-AuNPs. L-Cys-AuNPs were shown to have the highest impact on periodontal-tissue regeneration in vivo testing using a rat periodontal-defect model. Cell differentiation and tissue regeneration may have resulted from autophagy being activated in L-Cys-AuNP-treated hPDLCs. L-Cys-AuNPs are more effective in cellular internalization, autophagy regulation, osteogenic differentiation of cells, and periodontal tissue regeneration than D-Cys-AuNPs [52].

PTEN-induced putative kinase 1 (PINK1) dependent mitophagy affects inter-clonal communication among PDL stem cells (PDLSCs) with osteogenic heterogeneity; however, the method by which AuNPs do this is unclear. After 24 h in the presence of 20 nm AuNPs, PDLSCs’ proliferative capacity was almost at its peak. AuNPs, which induced PDLSC proliferation and osteogenic differentiation, also improved mitochondrial quality and homeostasis and resulted in a decrease in the number of mitochondria. PDLSCs also increased mitophagy. PINK1 activation enhanced mitophagy, mitochondrial quality, homeostasis, and osteogenic differentiation in PDLSCs through AuNPs in a synergistic manner [71].

In clinical settings, cell sheet technology (CST) is useful for mending alveolar bone abnormalities, and osteogenic stimulation before implantation may increase bone regeneration. By increasing the production and mineralization of bone-related proteins, AuNPs may support PDLSC sheets’ osteogenic development. In comparison to 13-nm AuNPs, the 45-nm AuNPs were more efficient. Additional research revealed that the activation of the autophagy pathway via the overexpression of microtubule-associated protein light chain 3 and the downregulation of sequestosome 1/p62 may be necessary for their capacity to induce differentiation. In addition, AuNPs considerably aided PDLSC sheets' bone repair in ectopic models [72]. In a different study, researchers discovered that AuNP treatment restored the autophagy-lysosome system, which had been impaired by inflammation, and thereby restored the osteogenic capacity of PDLSCs cultured under inflammatory conditions. In this situation, the downfall of TFEB in PDLSCs rendered AuNP ineffective. Studies show the critical importance of the autophagy-lysosome system in cellular osteogenesis under inflammatory circumstances, and they also point to a novel target for restoring inflammation-induced cell dysfunction employing nanomaterials to support cell biology and tissue regeneration [73].

#### Gold nanoparticles as a photothermal therapy agent in periodontitis

Typically, visible light is employed as the primary illuminating source to activate the photosensitizer in the context of periodontal therapy. The utilization of red light as the source of excitation is preferred due to its ability to penetrate more deeply compared to lasers emitting violet, blue, green, or yellow light. Nevertheless, the restricted ability of visible light to penetrate deeply hinders its capacity to effectively disrupt or eliminate periodontal pathogens within the deep periodontal pocket. The near-infrared (NIR) light encompasses a biological tissue window spanning from 700 to 1100 nm. The utilization of NIR light for excitation purposes offers enhanced tissue penetration capabilities and reduced levels of autofluorescence, thereby mitigating the potential side effects of phototoxicity and photodamage [74]. AuNPs exert good photothermal and antibacterial properties under NIR irradiation [75, 76]. AuNPs have garnered considerable interest as a result of their localized surface plasmon resonance (LSPR) when exposed to light. The absorption band of photosensitizers in LSPR leads to a significant increase in the absorption rate of the photosensitizers due to the presence of a high instantaneous electric field. Subsequently, this phenomenon has the potential to result in an elevation of the photosensitizer excitation rate, consequently augmenting the generation of ROS. The photoinactivation efficacy of methylene blue (MB)-conjugated Au nanoparticles (MB-AuNPs) was found to be highly effective in combating four-day-old biofilms of methicillin-resistant *Staphylococcus aureus* (MRSA). When subjected to irradiation, the biofilm experienced a reduction of more than 5 log10 when treated with MB-AuNPs, whereas the reduction was less than 1 log10 when treated with MB alone. Hence, the strategy of conjugating photosensitizers onto the surface of AuNPs proved to be a highly effective method for enhancing the efficacy of antibacterial photodynamic therapy [77, 78]. In a separate investigation, the synthesis of core–shell nanostructured Au nanorods/SiO2 with embedded verteporfin (AuNRs@SiO2-VP) was conducted to employ antibacterial photodynamic therapy against *Escherichia coli* (*E. coli*). After subjecting the sample to multiple rounds of irradiation, it was observed that a concentration of 104 CFU/mL of *E. coli* was effectively eliminated using AuNRs@SiO2-VP NPs at a wavelength of 710 nm (1 W/cm2). Furthermore, the duration of irradiation and the specific wavelength of the laser was found to have a significant impact on the efficacy of the nanostructures in combating pathogens [77, 79].

**Fig. 3:**
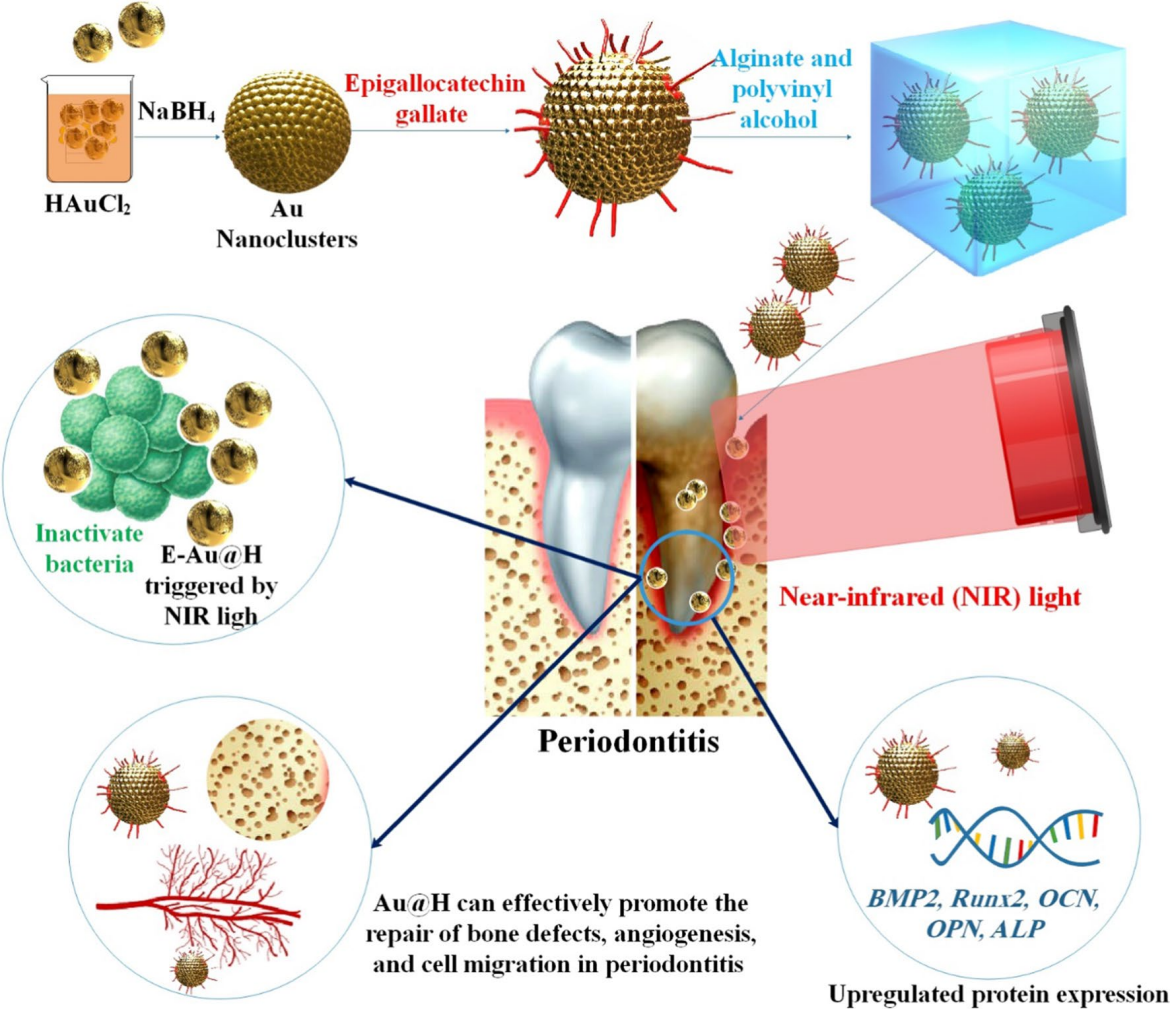
To treat periodontitis, the composite nanoplatforms of E-Au@H activated by NIR light have been shown to promote angiogenesis, cell migration, osteogenesis, and antimicrobial effects. The ability of E-Au@H to stimulate bone healing in periodontal infection models was proven in vitro. More importantly, the trials confirmed the composite's biosafety. Investigator’s work shows the promising effects of E-Au@H on periodontitis models and provides a novel approach to treating periodontitis [53]

Hydrogels coated with AuNPs (E-Au@H) were loaded with epigallocatechin gallate (EGCG) to promote NIR photosensitization, antibacterial activity, and periodontal tissue regeneration. Under NIR light irradiation, E-Au@H quickly warms up to 50.7 °C within 5 min, exhibiting a good photothermal impact; moreover, the NIR spectrum may effectively regulate the release of tea polyphenols, increase the antibacterial effect, stimulate angiogenesis, and boost bone regeneration. NIR-irradiated composites suppressed *S. aureus*, *E. coli*, and *S. aureus* biofilms by 94%, 92%, and 74%, respectively, according to in vitro experiments. They also raised ALP activity fivefold after seven days and the rate of extracellular matrix mineralization thrice after 21 days. The E-Au@H with NIR light irradiation efficiently suppressed dental plaque biofilm by 87% and accelerated alveolar bone regeneration by 38% in the rat periodontitis model. According to the findings of the experiments, this hydrogel with composite nanoplatforms that are NIR-sensitive provides new opportunities for the treatment of periodontal disease [53]. The detailed investigation protocol of E-Au@H in the treatment of periodontitis is demonstrated in Fig. 3.

**Fig. 4:**
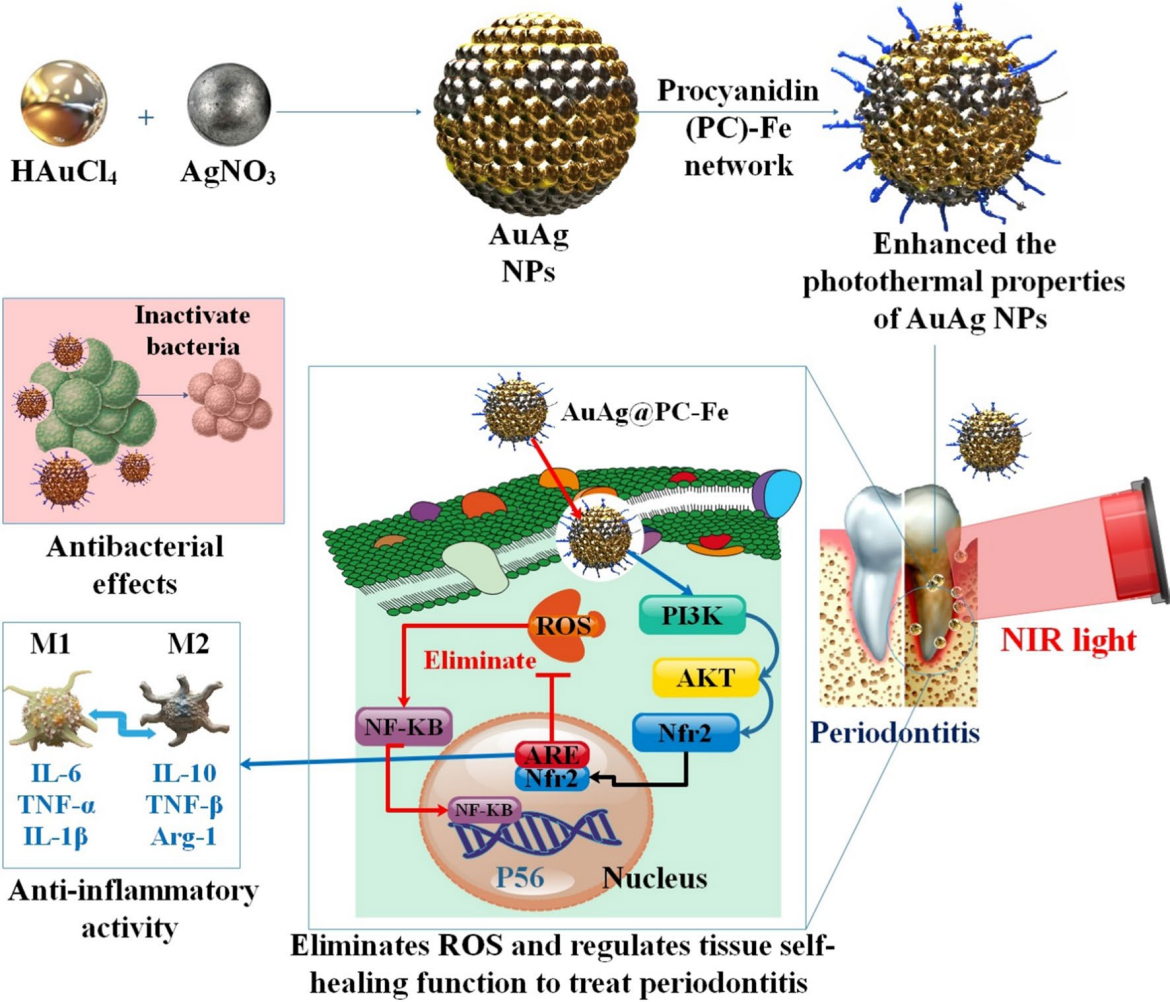
When stimulated by NIR light at 808 nm, the AuAg@PC-Fe quickly eliminates periodontal bacteria. Eliminating ROS and controlling tissue self-healing function through the PI3K/Akt signaling pathway is how AuAg@PC-Fe treats periodontitis. A novel and intriguing approach to treating periodontitis and other infectious inflammation is to investigate the use of MPNs to enhance the photothermal characteristics and anti-inflammatory action of materials [15]

Metal-phenolic networks (MPNs) are multifunctional hybrid nanomaterial nanoplatforms formed by the coordination of metal ions with polyphenols, and they have the potential for wide-ranging medicinal applications. AuAg@PC-Fe NPs, also known as branched AuAg NPs, were thus stuffed with MPNs. In addition to reducing oxidative stress and inflammation, the procyanidin (PC)-Fe network improved the photothermal characteristics of AuAg NPs, leading to more effective photothermal antibacterial activity against periodontal pathogens. To control immunity, AuAg@PC-Fe activates the phosphoinositide 3-kinase/protein kinase B signaling pathway, upregulates nuclear factor erythroid 2-related factor 2, scavenges ROS, and then inhibits the nuclear factor kappa-B signaling pathway. The capacity of inflamed periodontal tissue to heal in vivo was enhanced. Using MPNs in photothermal treatment and immunotherapy are two potential new applications inspired by this concept. It offers a novel treatment foundation for combating periodontitis and other infections. As depicted in Fig. 4, AuAg@PC-Fe can quickly eliminate periodontal pathogens under 808 nm NIR light excitation. The mechanism by which AuAg@PC-Fe removes ROS and controls tissue self-healing role to treat periodontitis is via the PI3K/Akt signaling pathway [15].

### Properties of silver nanoparticles

Pure crystalline Ag is used to make AgNPs ranging from 1 to 100 nm. Due to their improved and distinct physicochemical features, such as smaller particle size, larger surface area, and quantum confinement effects, they have lately acquired favor for a wide variety of biological applications [80, 81]. As a result of their ability to stop bacteria from producing NPs, oxides, and biofilms, AgNPs are often employed in the dentistry sector. They also block bacteria's metabolism. Dentistry must adapt to evolving patient requirements and cutting-edge technologies. AgNPs may be used in dentistry to clean the mouth and stop infections from occurring. AgNPs have shown promise in the dentistry sector for the removal of plaque and tartar as well as the removal of bacterial and fungal diseases in the mouth [82]. In addition, AgNPs are used in the disciplines of implantology, prosthodontics, endodontics, restorative dentistry, orthodontics, and periodontics. Due to their advantageous antibacterial qualities, they have mostly been employed for oral infection prophylaxis, disinfection, and prevention [83]. Additionally, to enhance their application in the treatment of periodontitis, collagen, a nontoxic, organic polymer, was used in the biosynthesis of AgNP solutions by Ag ions reduction [84]. For instance, one of the main causes of dental restoration failure is secondary caries, which often develops as a result of acids created by biofilms at the tooth-restoration bonded contact. This study's goals were to (1) create nanosized EMT zeolites with Ag ion exchanging; (2) add them to a commercial dental adhesive; and (3) look into how to prevent the growth of biofilms at the edge of tooth restoration. The template-free zeolite nanocrystals are produced, stabilized in water suspensions, and immediately put to use in the exchange of Ag ions. To create single-species biofilms, the cariogenic pathogens *Streptococcus mutans* (*S. mutans*), *S. gordonii*, and *S. sanguinis* were chosen as representative strains. The color and aesthetics of the tooth repair would not be compromised by the use of Ag + -EMT zeolites. Early attachment, bacterial biomass, biofilm formation, and metabolic activity were all significantly reduced by the EMT zeolites with the longest exchanging times (40 min) in the adhesives. All three cariogenic infections showed a nearly 2-order-of-magnitude decrease in the number of biofilm Colony-forming Units (CFU). As a result, the “bioactive” adhesive materials made from Ag + exchanged zeolites displayed outstanding antibacterial capabilities that demonstrated their promise for anti-biofilm and anti-caries clinical applications [85].

#### Silver nanoparticles in periodontitis treatment

Researchers studied biosynthesizing AgNPs utilizing Oroxylum indicum (L) Kurz (OI) stem bark extracts as a reducing agent (OI/AgNPs) and analyzed the biological effects of OI/AgNPs on hPDLSCs. With a size range of 21.49 0.32 nm, the OI/AgNPs were stable spherical NPs, and biosynthesis improved their biological and antioxidant activities. It was shown that hPDLSCs with high levels of cell viability accumulated OI/AgNPs in their cytoplasm. OI/AgNPs reduced the amounts of IL-1b secretion from LPS-hPDLSCs and boosted the cell proliferation of H2O2-hPDLSCs. The hPDLSCs' ALP activity and calcium content were both elevated by the OI/AgNPs. The biosynthesized OI/AgNPs were noncytotoxic, capable of shielding hPDLSCs from oxidative stress and inflammatory stimuli, and promoted osteoblastic development; as a result, they can use for the regenerative therapy of peri-implantitis [86].

**Fig. 5:**
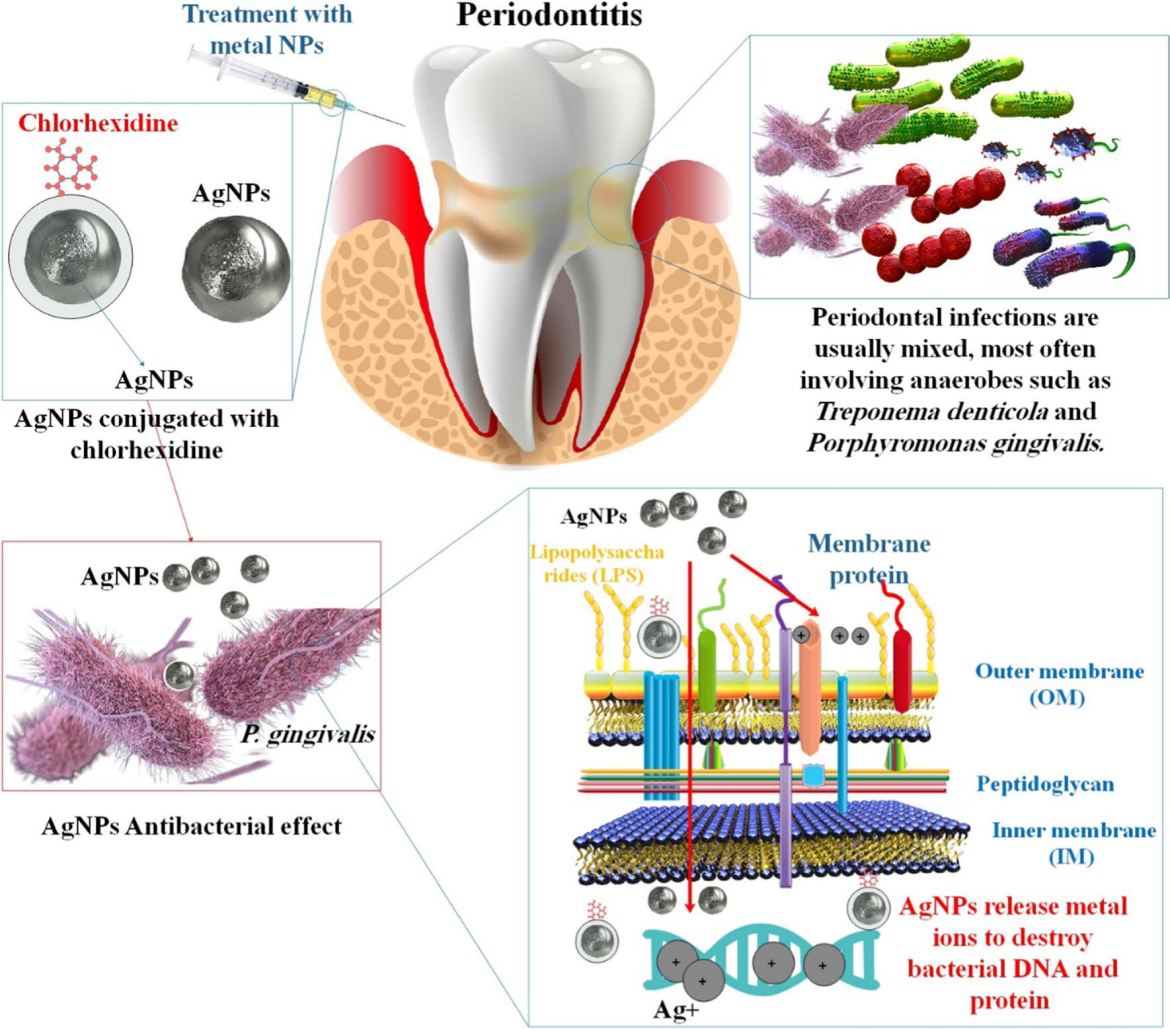
Mechanisms of antibacterial activity for AgNPs are diagrammatically representation in periodontitis. AgNPs and released Ag + ions act as efficient antibacterial factors. AgNPs-CHX may be possible therapeutic options for periodontitis, as they have antibacterial and anti-inflammatory properties

An Ulvan-based AgNP system’s antibacterial performance was assessed in another investigation. Ulvan, a polysaccharide isolated from Ulva lactuca and sulfated, was used in the environmentally friendly production of biogenic AgNP. AgNPs were used to create a new mouthwash, which underwent effectiveness and safety testing. The characterization of the AgNPs was established using Fourier transform infrared spectroscopy (FTIR), X-ray diffraction (XRD), and transmission electron microscopy (TEM), and the AgNPs were validated using spectrophotometric analysis (UV-A visible spectrophotometer). The Diploma in Dental Public Health (DDPH) assay was used to perform the antioxidant experiment, and at a concentration of 50 L/mL, AgNPs demonstrated 93.15% inhibition. At a concentration of 100 L/mL, the mouthwash containing AgNPs showed antibacterial efficacy against *Lactobacillus*, Candida *albicans* (*C. albicans*), *S. mutans*, and *S. aureus*. This research demonstrates that mouthwash made using the Ulvan-AgNP technology may be a safe and efficient oral antibacterial agent [54]. Another work used endophytic fungi to biosynthesize, characterize, and test the antibacterial effectiveness of AgNPs against *P. gingivalis*. AgNPs were characterized using the endophytic fungus *Fusarium semitectum*, visual inspection, UV–Vis spectrophotometer, TEM, SAED, and FTIR, and the agar diffusion technique was used to assess the antibacterial effect against the periodontal pathogen *P. gingivalis*. The manufacture of AgNPs using biological components such as bacteria, fungi, herbal extracts, and yeasts is a relatively new technique. Several antibiotics have been effective in combating periodontal infections. To treat periodontal disease, CHX is the most often utilized antibiotic. In the present research, biosynthesized AgNPs were employed as an alternative to topical antiseptics and antimicrobial agents for periodontal treatment; they may be mixed with other antimicrobial agents to provide a synergistic effect and are used for local medication administration [55]. The antibacterial properties of CHX and AgNPs have been shown against a wide range of periodontal and oral infections. The PerioChip is a CHX digluconate-containing biodegradable gelatin that has already hit the market for controlled release. In tissue engineering, where the existence of porosities is crucial for cell adherence and cell proliferation in tissue formation, polymer hydrogels have also been employed as a scaffold. Hydrogels based on poly (2-hydroxyethyl methacrylate) and doped with AgNPs have shown promising antibacterial activity against *E. coli* and *S. aureus* in vitro. Ag ions were shown to have a variety of effects on bacterial cell structures, including an increase in membrane permeability that ultimately results in cell lysis. DNA, bacterial proteins, and lipids are all vulnerable to damage by AgNPs. When used at a low enough concentration, AgNPs do not affect cell differentiation but do enhance cellular stress and toxicity. An essential step forward in the treatment of periodontal disorders and peri-implantitis would be the creation of a porous hydrogel-based drug-delivery system that could also serve as a scaffold for tissue regeneration [87]. In Fig. 5, we have summarized the antibacterial properties of AgNPs on bacteria associated with periodontitis.

### Zinc nanoparticles

Because of their antibacterial capabilities, ZnONPs have become one of the most commonly utilized nanoparticulate materials in the world. Therefore, understanding the mechanism of action (MOA) of ZnONPs' bactericidal properties might aid in the development of prediction models for countering bacterial resistance [88]. Dentin remineralization and tubular occlusion may be accomplished with the help of Zn-doped NPs. In endodontically treated teeth, this provides a novel approach to regenerating degraded cervical dentin and effectively treating dentin hypersensitivity before canal filling. Zn NPs' antimicrobial capabilities also aid in reducing biofilm development [89]. ZnONPs are effective against microorganisms. Flowable composite resins were tested for their abrasion resistance, transparency, and microhardness after being treated with various concentrations of these NPs and associated chemical and physical mixes [90]. An effective antibacterial agent, ZnO works via several processes involving several chemical species. In the literature, three main modes of action have been proposed: The semiconductive nature of ZnO causes (i) the generation of ROS, (ii) the destabilization of microbial membranes upon direct contact between ZnO particles and the cell walls, and (iii) the inherent antibacterial capabilities of Zn2 + ions produced by ZnO in aqueous media [91]. ZnO NPs were discovered to have a distinct antibacterial inhibitory impact on gram-positive and gram-negative bacteria strains. According to many research, gram-positive bacteria were more often inhibited than gram-negative bacteria [92]. As an illustration, the bacterial strains *E. coli*, *P. aeruginosa*, *Bacillus subtilis* (*B. subtilis*), and *S. aureus* were subjected to ZnONPs at concentrations corresponding to their IC100. This exposure was carried out in 100 μL of media for a duration of 15 min. ZnONPs can adhere to the surfaces of both Gram-positive and Gram-negative bacteria via distinct mechanisms. The presence of teichoic acid within the peptidoglycan layer and lipoteichoic acid within the cell membrane contribute to the generation of negative charges on the surface of the cell. The electrostatic interactions between the positive charges of ZnONPs and the cell surface result in their attraction, ultimately causing damage to the cell surface due to the disparity in electrostatic gradients. Teichoic and lipoteichoic acids function as chelating agents for Zn2 + ions, facilitating their transportation through membrane proteins via passive diffusion. Furthermore, bactericidal activity can manifest through various mechanisms, including adsorption onto the bacterial surface, generation of diverse intermediates, and electrostatic interactions [93, 94]. Metal NP photocatalysts have recently received attention due to their high ability to absorb ultraviolet and visible light. ZnONPs, one of the antimicrobial photo-sonosensitizers employed in this approach, produces ROS when subjected to light and ultrasound vibrations. Compared to Ag and other NPs, ZnONPs are more biocompatible. But given the intrinsic toxicity of the metal and the solubility of NPs defined by the metal's chemical characteristics, absorption, and capacity to cause oxidative stress, it may still be deadly at large levels. The use of lesser concentrations, on the other hand, reduces its antibacterial capabilities. When ZnONPs are exposed to light (365–400 nm) or ultrasonic waves, ROS are produced, which causes oxidative stress and improves antibacterial capabilities at low concentrations. The objective of this study was to assess the efficacy of ROS-based antimicrobial approaches on polymicrobial periopathogenic biofilms consisting of *P. gingivitis*, *P. intermedia*, and *A. actinomycetemcomitans*. These biofilms were formed on mini-screws that were coated with ZnONPs. Additionally, the study aimed to investigate the impact of these antimicrobial strategies on the expression levels of inflammatory factors (TNF-α, IL-1β, and IL-6) in human gingival fibroblast (HGF) cells surrounding the mini-screws. The study revealed that the utilization of ZnONPs in antimicrobial photo-sonodynamic therapy resulted in a substantial reduction in periopathogen biofilm formation, along with a notable decrease in the expression of inflammatory cytokines [95].

ZnNPs have an absorption peak between 230 and 330 nm. ZnNPs ranged in size from 30 to 80 nm, with the majority having a size between 50 and 60 nm. ZnNPs + CHX was reported to have a Minimum inhibitory concentration (MIC) of 1.66 g/ml against *S. mutans* and 1.66 g/ml against *Actinomyces viscosus* (*A. viscosus*). Furthermore, 13.33 and 16.33 g/ml for *S. mutans* and *A. viscosus*, respectively, were found as the lowest MICs associated with ZnNPs alone. Both bacteria’s growth was significantly slowed by ZnNPs + CHX compared to CHX (P 0.05) [96]. It is believed that ZnNPs bio-fabricated from renewable sources are safe for the environment [7]. Brackets may be used to prevent the spread of *S. mutans* during orthodontic therapy if they are coated with CuO and ZnONPs [37].

Carbopol 940® hydrogels were modified to include prepared ZnONPs. The hydrogel's therapeutic benefits and the gingival tissue’s capacity for self-repair are more noticeable than those of 2% minocycline ointment (Perio^®^). When the ZnONPs content was higher than 0.2 g/mL, the hydrogel also exhibited broad-spectrum antimicrobial and efficient antibacterial capabilities. More than 85% of cells were still alive after being exposed to ZnONPs at concentrations lower than 0.8 mg/L, demonstrating their low toxicity and good security. The Mino-ZnO@Alb nanohydrogel, in conclusion, demonstrated exceptional multifunctional capabilities, such as pH responsiveness, broad antibacterial range, sustained release, tissue-repairing, and adhesion qualities. Mino-ZnO@Alb hydrogel, in comparison to Perio^®^, may be a promising solution since it may boost bioavailability, cut down on minocycline dose, lessen adverse effects, and boost patient compliance. A good and appropriate choice for regulated drug administration through bio-adhesive hydrogel may be the combination of metal oxide NPs. This method may enhance stability and serves as an illustration of specialized nanocarriers for better medication delivery [97]. The synthesized ZnONPs are spherical and have molecules with a size of around 80 nm. The impact of ZnONPs on Alanine Transaminase (ALT) activity was examined in the saliva of 20 patients with chronic periodontal disease and 15 healthy individuals. The results showed a considerable rise in ALT activity when ZnONPs were used. According to Rai et al., the ALT level in saliva has a significant clinical significance as a marker of periodontitis since it might represent the degeneration and inflammation of periodontal tissue. The results showed that individuals with chronic periodontitis had salivary ALT activity as compared to control subjects and that the activity of ALT was affected by ZnONPs when compared to the same chronic periodontitis patients without conclusion. ZnONPs stimulate the activity of the ALT enzyme [98]. PolymP-nActive NPs loaded with calcium or Zn. In calcium-loaded NPs, calcium and phosphate precipitate on the NP surfaces. In all experiments, it was discovered that non-loaded NPs were not hazardous; nevertheless, calcium and Zn-loaded particles showed a dose-dependent but extremely modest cytotoxic impact. In addition to their apparent non-toxicity, calcium-loaded NPs' capacity to encourage the formation of calcium phosphate deposits may provide novel approaches for treating periodontal disease [99]. Infection of the exposed membranes and postoperative problems may be avoided by improving bacterial resistance when designing membranes for guided tissue regeneration (GTR) to treat periodontal disorders. This research added antibacterial ZnONPs to a biocompatible and biodegradable substance like polycaprolactone (PCL) to assess the antibacterial activity of PCL/ZnO membranes and their impact on cell survival. Bacteria like *S. mutans* and *P. gingivalis* were tested for their ability to adhere using a crystal violet test. The ZnONPs were dispersed throughout the PCL matrix of the PCL/ZnO membranes. When compared to pristine PCL membranes, these have a diminished ability to form crystals and an amplified amorphous shape. Compared to the clean PCL membranes, the adherence of *S. mutans* and *P. gingivalis* was drastically reduced in the LZ and HZ groups containing ZnO. The results of this investigation show that PCL membranes loaded with ZnONPs limit bacterial adherence without compromising osteoblast viability, indicating that ZnO may be used in GTR to boost the antibacterial activity of membranes [58].

As anticipated, the membrane coated with ZnoNPs effectively exhibited bactericidal activity against the periodontitis-causing bacteria. ZnoNPs exhibited antibacterial efficacy against various bacterial strains, including *S. aureus*, *S. mutans*, *P. gingivalis*, and *Fusobacterium nucleatum* (*F. nucleatum*). Notably, the presence of *F. nucleatum* species resulted in an improved and more predictable periodontal regeneration outcome. Although ZnONPs are thought to be a biologically safe material that does not display cell toxicity, there is still a need for further discussion, questioning, and study into the regulatory and safety issues in dental care products on long-term usage. Most of the investigations done on these NPs have been conducted in vitro, and there have been very few studies done on animals. Therefore, further research and clinical trials are needed to fully realize its potential [100–102].

### Titanium dioxide nanoparticles

NPs of titanium dioxide (TiO2NPs) have sizes of fewer than 100 nm. TiO2NPs, like many other types of NPs, have distinct surface chemistry and morphologies. TiO2NPs have also been widely used for their photocatalytic, antibacterial, and antiparasitic properties, making them stand out among metal oxide NPs [103, 104]. Due to their high refractive index, adequate chemical stability, cheap cost, and powerful oxidation capabilities, TiO2NPs have become a widely used and widely produced oxide [105]. Since TiO2NPs’ photocatalytic activities create free radical oxides and peroxide with powerful antibacterial activity and wide reactivity against various microbial pathogens, they also showed to have a strong antimicrobial impact. TiO2NPs are more effective against Gram-negative bacteria (like *E. coli* and *P. aeruginosa*) due to their thinner cell wall; however, they are less effective against Gram-positive bacteria (like *S. aureus* and *Enterococcus faecium*) and yeast species (like *C. albicans*) with thicker cell walls [106]. A typical paradigm among them that might react to ultrasonic irradiation to create ROS for antibacterial therapy is TiO2 with acceptable biocompatibility. Due to its narrow band gap and rapid recombination of electrons (e−) and holes (h +) when exposed to ultrasound, pure TiO2NPs exhibit a low quantum yield of ROS [49]. Due to its biocompatibility, biosafety, and lack of allergic responses with human tissues, TiO2NPs are suitable for use in cutting-edge medical and dental applications. Until recently, it was believed that their biocompatibility was determined mostly by their dimensions, shapes, phases, surfaces, coatings, and topographies. Recent studies on TiO2NPs have shown that the way of synthesis is the primary reason why these NPs are biocompatible, which is why they are increasingly being used in medical and dentistry settings [107]. One of the primary causes of periodontitis is resistance to TiO2NPs' potent bactericidal and sterilizing actions. Because of their exceptional photocatalytic activity and chemical stability, TiO2NPs are suitable additions for boosting the characteristics of polymer materials used to treat periodontitis [108, 109].

The risk of peri-implantitis infections may be raised by periodontopathogen bacteria attaching to intraoral components of implants that are exposed to saliva, plaque, and crevicular fluid. To demonstrate the potential clinical value of Ag-doped TiO2 nanotubes for providing antimicrobial properties against the adhesion of peri-implantitis-associated bacteria *A. actinomycetemcomitans*, *Tannerella forsythia*, and *Campylobacter* rectus for transmucosal components of dental implants, the goal of this study was to indicate the potential clinical benefit of these nanotubes. During annealing, the concentration of Ag in TiO2 nanotubes was significantly elevated. When tested against *A. actinomycetemcomitans*, *T. forsythia*, and *C. rectus*, as-annealed Ag-doped TiO2 nanotubes were shown to be potent antibacterial agents [59]. Some cells produce salivary ALP, an enzyme that is attached to the cell membrane. The oxidation processes of various compounds are catalyzed by peroxidases, which are oxidizing enzymes. Active periodontal disease is associated with an increase in salivary ALP and peroxidase activity. Salivary ALP and peroxidase activity are increased in individuals with chronic periodontitis, and TiO2NPs may be responsible [110]. Scientists looked into the molecular reasons behind the pro-inflammatory action of TiO2NPs on human PDL cells and determined whether or not they stimulate cyclooxygenase-2 (COX-2). Both the mRNA and protein levels of COX-2 were increased when PDL cells were treated with TiO2NPs. TiO2NPs induced phosphorylation and subsequent degradation of the inhibitory protein IB in PDL cells, which promoted nuclear translocation of nuclear factor-kappaB (NF-kB) and its DNA binding. Rapid activation of ERK1/2 and Akt, which may be upstream of NF-κB, was seen after treatment with TiO2NPs. TiO2NPs generated NF-κB activation and COX-2 expression were significantly suppressed in PDL cells by treatment with the MEK1/2 inhibitor U0126 and the PI3K inhibitor LY294002. There was an increase in intracellular ROS formation in PDL cells after treatment with TiO2-NPs. In conclusion, the inflammatory impact of PDL cells may be at least partially attributed to the ROS, which is simultaneously overproduced by TiO2NPs and stimulate COX-2 expression via activation of NF-κB signaling [111].

Antibacterial photodynamic treatment using nanomaterials has been reported very seldom, however, the nanosurface layer has shown promise in preventing peri-implantitis. Ag plating, anodization, and sintering were used to make nanocoatings of Ag, titanium dioxide, and hydroxyapatite (HA) for implants. The biofilm on the surface of the implants was decreased by 97.5% and bacterial growth in the surrounding medium was entirely suppressed thanks to the Ag -nHA nanocoating. Reduced infection risk, improved osseointegration, and faster bone healing were all benefits of this innovative nanocoating for titanium alloy implants [77]. As a result of its high stability and strong photocatalytic activity, TiO2 is frequently utilized as a catalyst. The ROS generated by photocatalytic TiO2 is very effective in killing and sterilizing oral microorganisms. TiO2 has a broadband gap energy of 3.2 eV, making its activation by ultraviolet (UV) light the sole viable option for its use. UV rays may also damage DNA and kill cells via irradiation. Upconversion nanoparticles (UCNs) have recently been produced to address this issue and find widespread use in cancer treatment. But antimicrobial and bacteriostasis have seen less study. The photocatalytic activity of TiO2 is activated when it is exposed to near-infrared light; the UCN core does this by converting the low-energy NIR light to high-energy UV light via an anti-Stokes emission mechanism. Recently, a core–shell structured NaYF4:Yb3 +, Tm3 + @TiO2 (TiO2-UCNs) was synthesized using this technique and used in aPDT against periodontal infections. Thermal breakdown in an oleic acid environment was used to create the NaYF4:Yb3 +, Tm3 + cores for UCNs. For surface modification of UCNs, polyvinylpyrrolidone (PVP) was applied. The TiO2 shell on the UCNs’ surface was then coated with titanium n-butoxide as a titanium precursor during hydrothermal processes. It was discovered that periodontal pathogens like *P. gingivalis* and *F. nucleatum* were no match for TiO2-UCNs’ potent antibacterial activity. The number of CFU of bacteria in the light groups was reduced by four to five orders of magnitude. When the medication concentration was 2 mM, the bacterial solution was almost eliminated 4 h after the first irradiation of 2.5 W cm^2^ for 5 min. Antibacterial photodynamic therapy induced by near-infrared light is therefore a potentially useful method for treating periodontitis [74, 112].

TiO2NPs are widely utilized in the food industry and are commonly included as components in various pharmaceutical products and cosmetics, including sunscreens and toothpaste. Despite the widespread utilization of TiO2NPs, there remains a lack of comprehensive understanding regarding their biological effects and the underlying cellular response mechanisms. Consequently, it is imperative to acquire a thorough comprehension of the toxicological characteristics of this substance. The creation of ROS seems to be the primary mechanism behind the potential for toxicity caused by TiO2NPs, which may lead to oxidative stress, inflammation, genotoxicity, metabolic alteration, and even the development of cancer. Size, crystal structure, and photo-activation of TiO2NPs are some of the chemical and physical factors that have a significant impact on the degree and kind of cell injury. To fully use it in the treatment of periodontitis, further research and clinical studies must be conducted [113].

### Magnetic nanoparticles

Due to their distinct physicochemical features and their capacity to operate at both the molecular and cellular levels, magnetic NPs (MNPs) have gained interest in the field of dental medication delivery with broad diagnostic and therapeutic applications. As a result, NPs and magnetic forces have been employed in dentistry in recent years to transport drugs for the prevention and treatment of tooth ailments. MNPs-based medicines are very successful against multidrug-resistant bacterial and fungal infections. The idea of MNPs has enhanced the transparency and wear resistance of dental materials used in cosmetic dentistry. Dental caries and endodontic and periodontal diseases have decreased because of the usage of MNPs in dental medication delivery systems of several types. Dental composites and adhesives containing MNPs have been shown to reduce biofilm development [114]. MNPs are composed of three distinct layers: a magnetic iron core, an inner layer of lipophilic fluorescent dye, and an exterior layer of polysaccharide or chitosan matrix [114]. The vast majority of NPs used in biomedical settings are iron oxide NPs (IONPs), namely magnetite (Fe3O4) and its oxidized counterpart maghemite (γ-Fe2O3). Cobalt (Co) and nickel, both highly magnetic compounds, are of little interest since they are poisonous and prone to oxidation [115]. Because of the unique physicochemical features intrinsic to the nanoscale, IONPs have garnered tremendous interest in a variety of applications. Iron oxides' versatility is expanding thanks to their size, surface area, quantum confinement, and unique magnetic and optical properties [116].

Dental plaque is a significant contributor to periodontal illnesses including gingivitis and periodontitis. Plaque biofilms may be controlled, and gingivitis and periodontitis can be prevented or treated, thanks to the inclusion of many antiplaque and anti-microbial compounds in tubes of toothpaste and mouthwashes. More research on MNPs has recently been conducted in the dental field. Drug transport via the oral mucosa may be enhanced by using MNPs, and MNPs can also improve the mechanical and microbiological qualities of dental prostheses and implants [117]. To eradicate the periodontal plaque and inactivate target bacteria, including *P. gingivalis*, standard antibacterial agents have a hard time entering the periodontal pocket directly due to the complexity of the oral environment. To address this issue, this research suggests using magnetic induction for targeted attachment of antimicrobial MNPs (AMPs) that can anchor poly hexamethylene biguanide (PHMB) to treat periodontitis. According to in vitro antibacterial investigations, AMPs may eliminate bacterial biofilms with a clearance rate close to 80% by using magnetic inductivity, the nano size effect of NPs, as well as the antibacterial impact of PHMB. More significantly, periodontitis-related in vivo investigations showed that gingival redness and suppuration vanish following AMPs therapy. According to the quantitative analysis of microCT, in contrast to the periodontitis groups, the AMPs groups showed evidence of alveolar bone development. Additionally, Hematoxylin and eosin (HE) staining supported the histological findings that the AMPs treatment group had increased alveolar ridge height and bone density but decreased inflammatory factor infiltration, supporting the therapeutic impact of AMPs on periodontitis. As a result, it is anticipated that the AMPs suggested in this research will be extensively used as drug carriers for the treatment of periodontitis [118]. In a different study, scientists added MNPs and the antibacterial compound dimethylaminododecyl methacrylate (DMADDM) to the EndoREZ root canal sealer. Researchers used magnetic fields to test the penetration depth of root canal sealant both in vitro and in vivo. According to the findings, EndoREZ sealer with 2.5% DMADDM and 1% MNP was biologically safe and capable of sealing the apex. Additionally, with an external magnetic field, the modified sealer might broaden its area of penetration and have a considerable antibacterial impact on multispecies biofilms. The in vivo investigation found that the sealer with 2.5% DMADDM and 1% MNP had a good inhibitory effect on persistent apical periodontitis because it prevented considerable resorption and only slightly increased the PDL space at the apices of root canals [119]. Another research will examine how IONPs impact rat collagenase-1 (COL-1) and ALP levels as well as periodontal disease. In this work, the levels of Col-1 and ALP activity in rats with periodontal damage were measured. By using HE staining, researchers were able to identify periodontal histological alterations as well as the expression of periodontal pocket depth (PD) and attachment loss (AL). By using a Western blot, they also discovered the proteins Col-1 and ALP in periodontal tissues. In the periodontal tissues of the rats in each group, RT-PCR identified the presence of Col-1 and ALP mRNA. As opposed to the sham group, the model group's gingival crevicular fluid had higher levels of ALP activity and Col-1 (P < 0.05). ALP activity and Col-1 content in the gingival crevicular fluid of model rats were reduced after IONP injection (P < 0.05). In the model group, the gingival atrophy was more severe, and many inflammatory cells invaded the tissue and damaged the alveolar tissue. While the PD and AL periodontal indices were considerably suppressed (P < 0.05), the periodontal tissue of the rats in the intervention group dramatically improved, and the degree of alveolar bone degradation was also significantly decreased. Protein and relative expression data showed that ALP and Col-1 levels in the periodontal tissue of the model group were significantly lower than those of the sham group (P 0.05). After receiving IONP, the intervention group showed enhanced mRNA and protein expressions of ALP and Col-1 in the periodontal tissues. Therefore, IONPs may suppress the production of ALP and COL-1 and improve the periodontal injured tissue in rats with periodontal damage [120]. Another study set out to do two things: (1) create multifunctional NPs comprised of Chlorin e6 (Ce6), Coumarin 6 (C6), and Fe3O4 NPs; and (2) look into the NPs' inhibitory effects via antibacterial photodynamic therapy (aPDT) on three different species of periodontitis-related pathogens. Superparamagnetic characteristics, chemical stability, water solubility, and minimal cytotoxicity were all exhibited by Fe3O4-silane@Ce6/C6 MNPs. The C6 and Ce6 emission peaks were not altered by the presence of Fe3O4 NPs. There was a statistically significant (p < 0.05) decrease in biofilms in the Fe3O4-silane@Ce6/C6-mediated aPDT groups compared to the control groups. Approximately 4–5 orders of magnitude reduction in CFU of biofilm was achieved using Fe3O4-silane@Ce6/C6-mediated aPDT. Ce6 and C6 co-loading allowed for continuous monitoring of aPDT through ratio emissions at a single wavelength. By combining a magnetic field with Fe3O4, we can pinpoint infection locations and eliminate germs in those areas. Strong anti-biofilm action was exerted by the multifunctional NPs against periodontitis-related bacteria, and the NPs were also biocompatible, able to be monitored in real-time, and could be targeted magnetically. The multifunctional NPs have much promise as an antibacterial treatment for preventing periodontitis [60].

### Nickel nanoparticles

Some research has demonstrated that nickel NPs (NiNPs) have antibacterial properties, and nickel is inexpensive enough for widespread application. Because of its high surface-to-volume ratio, nano-Nickel, like other nanomaterials, has greater contact with microbes and more activity. However, nickel is often utilized in dental compounds to improve the success rate of root fillings. The incidence of clinically significant adverse responses, such as contact allergies, to nickel as one of the dental metals is very low, as shown by several studies [121]. *S. epidermidis* was the most common specie in the oral cavity (27.27%) and periodontal pocket (15.9%). Higher percentages of non-resident bacteria found in subgingival samples and oral locations may pose a serious challenge in developing and sustaining periodontal diseases [122, 123]. In a few investigations, NiNPs were shown to exhibit anti-microbial properties against bacteria like *S. aureus* and *E. coli*. In this investigation, researchers used *S. epidermidis* clinical isolates to test the hemolytic impact of NiNPs on human red blood cells (RBC) and the inhibitory effect of NiNPs on biofilm development. All S. epidermidis clinical isolates were shown to be capable of biofilm generation in this investigation. The production of biofilm was shown to be suppressed by NiNPs. The coagulase-negative staphylococci group includes *S. epidermidis*, a Gram-positive coccus. It is the main source of infection in artificial heart valves, prosthetic joints, vascular grafts, intra-cardiac devices, and cerebrospinal fluid shunts. The ability to form biofilms, polysaccharide intercellular adhesion (PIA), biofilm-associated protein, poly-glutamic acid (PGA), staphylococcal enterotoxin-like toxin L (SEIL), and C3 enterotoxin (SEC3), phenol-soluble modulins (PSMs), Clpxp, and extracellular matrix-binding protein are all examples of virulence factors [124]. Biofilms are multicellular, surface-attached collections of bacteria that exhibit unique physiologic and architectural features, making them resistant to several types of antibiotics, including penicillin, aminoglycosides, quinolones, and various host defense mechanisms. The concentration of antibiotics needed to kill bacteria in a biofilm is one thousand times greater than the concentration needed to kill the same bacterium in a planktonic form [125]. However, NiNPs' potential for inhibiting biofilm formation has never been studied. This research set out to determine whether Ni-NPs might inhibit biofilm formation in *S. epidermidis* from a clinical setting. Furthermore, the equivalent hemolytic impact on human RBC was examined to ascertain the dangers of NiNPs for systemic application in live beings. While NiNPs' antimicrobial efficacy has been shown elsewhere, their potential to disrupt biofilms has not yet been explored. In patients with catheters or other surgical implants, *S. epidermidis* is a prevalent source of hospital-acquired infection. *S. epidermidis’s* ability to form biofilm is likely a contributing element to its infectiousness. Since *S. epidermidis's* capacity to form biofilms on viable and non-viable surfaces, notably on plastic devices, is its key virulence factor, the current research aimed to ascertain whether or not NiNPs might suppress the biofilm formation of *S. epidermidis* clinical isolates [61]. A novel Ni(II)-containing coordination complex has been successfully created by researchers via a solvothermal process. According to the findings, the substance significantly inhibits the growth of *P. gingivalis* biofilm. In conclusion, the substance effectively combats periodontal disease by preventing the growth of *P. gingivalis* biofilm [126]. The commonly used standard human pathogens *S. aureus* and *E. coli* were utilized to investigate the antibacterial activity of copper (Cu), Ni, and bimetallic Cu-Ni NPs. These NPs were further examined against the dental pathogen *S. mutans*. When it comes to *S. aureus*, *E. coli*, and *S. mutans*, CuNPs demonstrate a bactericidal impact whereas NiNPs and bimetallic Cu-Ni NPs only have a bacteriostatic effect. Cu-Ni-NPs are anticipated to demonstrate unique effectiveness against periodontitis-related bacteria or to prevent the production of biofilm [127].

### Bismuth nanoparticle

Despite the well-documented therapeutic usage of the bismuthcomplex, very few investigations have focused on the medicinal uses of bismuth NPs (BiNPs). Bactericidal, fungicidal, antiparasitic, and antibiofilm characteristics of metallic bismuth NPs have also been investigated. Toxicological data on these NPs have been acquired, however, they are not yet adequate to warrant their use in human clinical trials [128, 129]. Numerous publications discuss the creation of non-metallic BiNPs such as bismuth oxide, bismuth sulfide, bismuth selenide, and bismuth telluride as well as its use in biomedicine [130]. Therefore, due to their unique features, Bi2O3 NPs may have uses in the medical, dental, and aesthetic fields. Among them are their affordability and scalability, high stabilization, chemical inertness, nontoxicity, compatibility with biological systems, and active characteristics [131]. The emergence of a new strategy for the treatment of dental caries, oral infection, and infection related to the oral cavity, such as endocarditis and septicemia, is crucial and essential due to the high side effect of routine drugs used for oral infection, low efficacy, drug resistance, and use as a mouthwash, which could cause sensitivity and have low efficacy. To get around this issue, researchers used BiNPs. A recent study suggests that BiNPs might replace existing drugs or be used as a mouthwash to treat oral infections due to their low MIC, good effectiveness, and cheap cost [132]. BiNPs’ ability to stop *S. mutans*, the bacteria responsible for most cases of tooth decay, from forming biofilms is one of its defining features. Due to the electrostatic interaction between a negatively charged cell membrane and the positively charged NPs, it has been shown that positive charges on the metal ion are essential for antimicrobial activity. DNA damage, changes in gene expression, and effects on membrane-bound respiratory enzymes are among the other negative effects of AgNPs that have been documented [133]. Bismuth combined with a lipophilic dithiol (3-dimercapto-1-propanol, BAL) in a 2:1 molar ratio strongly inhibited the production of EPS by *Brevundimonas diminuta* (*B. diminuta*) in suspended cultures at concentrations slightly below the MIC. After 5 days of exposure to bismuth-BAL chelate (BisBAL) at near MIC (12 M), total polysaccharides and proteins released by *B. diminuta* reduced by nearly 95%. The suppression of carbohydrate O-acetylation was hypothesized to be one mechanism by which BisBAL disrupts biofilms using Fourier-transform infrared spectroscopy (FTIR). FTIR analysis also showed striking similarities between EPS samples treated and untreated with BisBAL, with protein, polysaccharide, and peptide expression levels being the main difference [134]. Nanomaterials have recently been used in the area of dentistry and medicine as a novel, alternative antibacterial agents. Bismuth subsalicylate (BSS) has been utilized as an antibacterial agent, but its potential efficacy against the bacteria that cause periodontal disease has not been specifically studied in the form of NPs (BSS-nano). This research aimed to analyze the safety of BSS-nano by analyzing their cytotoxicity in HGF-1 cells and the antibacterial efficacy of BSS-nano against oral anaerobic bacteria. It was shown that BSS-nano has a polygonal form and a main size of 4–22 nm. Dental materials and antiseptic solutions may use BSS-nano as an antibacterial agent [135]. Zerovalent BiNPs’ antibacterial effects have been the subject of preliminary but encouraging studies. They were just as successful in halting the spread of *S. mutans* as CHX. It is critical to keep in mind that zero-valent BiNPs have a 0.5 mM MIC for the inhibition of bacterial growth when thinking about including them in mouthwash. In the tests that have been done, CHX, the industry standard for oral antiseptics, has been demonstrated to have similar effects to these NPs. The introduction of zerovalent BiNPs completely stopped *S. mutans* from producing biofilm. This result was surprising since zero-valent BiNPs were anticipated to have an inhibitory effect on cell growth but not a complete block. Researchers hypothesized that because 69% of the cells were rendered inactive by NPs, there wouldn’t be enough cells left to form a biofilm. The majority of the experimental evidence points to these NPs as a potential treatment for bacterial infections based on biofilms [62].

To create innovative medicinal molecules with antibacterial capabilities for the treatment of various mouth illnesses, such as periodontal disorders, nanotechnology is now helpful. In this study, the laser ablation of solids in liquids (LASL)-created BSS-NPs were tested for their ability to kill bacteria on samples of subgingival biofilm from patients with periodontal disorders. The findings of the characterization demonstrated that both the triclinic crystallographic structure and the vibrational modes of the BSS functional groups were preserved in the synthesized BSS-NPs made by laser ablation of solids in liquids. When exposed to BSS-NPs, more than 30% of the total number of cultivable bacteria from the oral biofilm samples were suppressed. Because of this, BSS-NPs have the potential to be utilized as a novel alternative antibacterial in the treatment of periodontal disorders, lowering the usage of antibiotics without a prescription and halting the rise of antibiotic resistance [136]. This study investigated the effects of BiNPs on *Enterococcus faecalis*, the bacteria responsible for most chronic root canal infections. All samples were first cultured in *Enterococcosel* broth. After incubation, the PCR test was repeated on the samples that had shown signs of growth on blood agar plates. Powdered NPs were dissolved in ultrapure water, and the spectrophotometer was used to calculate the final concentration of BiNPs. The microbroth dilution technique was used to evaluate the MIC of BiNPs against *E. faecalis* by standard procedures for antimicrobial susceptibility testing. The quantity of BiNPs that resulted in a 99.9 percent reduction in the number of live bacteria was also determined using bactericidal experiments performed in the Mueller–Hinton broth medium. In light of the benefits of BiNPs, such as their ability to prevent biofilm formation from *S. mutans* and their greater antibacterial activity compared to CHX, it is suggested that they be used in a variety of dental settings to combat *E. faecalis* [63].

BiNPs have been shown effective in the treatment of *H. pylori* ulcers in people, and their antimicrobial properties have been demonstrated in several laboratory trials. One study (NCT04209933) aims to compare the safety and efficacy of three different forms of Bi (pectin BiNPs, Bi potassium citrate, and pectin Bi capsules) for the first treatment of *H. pylori* infection [137]. Since BiNPs have more antibacterial action and fewer adverse effects than CHX, they may be a more appealing choice to employ in various dental settings in the fight against *S. salivarius* and *E. faecalis*. BiNPs, however, need in-depth research. More extensive research with a bigger sample size is needed in this area. More research is needed to determine the toxicity and short- and long-term impact of these NPs on live cells [132]. Although BiNPs show promise as a means of preventing several infectious illnesses, they still need further testing to guarantee their safety for use in people. Over the last century, there have been reports of human Bi intoxication and even fatalities associated with the use of Bi pharmaceuticals. A hazardous dosage of Bi compounds causes acute renal impairment. Bi encephalopathy was a treatable neurological condition that emerged in the 1970s among patients who had taken excessive amounts of Bi over extended time periods. Since many people who had taken large amounts of Bi had not experienced these symptoms, the dose–response relationship between Bi intake and these symptoms remains unexplained. BiNPs' dose must also be carefully examined. Hence, it is imperative to conduct additional investigations to ascertain the potential cytotoxicity of BiNPs to identify any detrimental impacts on human health. The underlying mechanism by which BiNPs operate remains inadequately comprehended. The absence of a reliable in vitro analysis technique, coupled with the complexities associated with the bacterial membrane, presents challenges in obtaining a comprehensive understanding of the precise antimicrobial mechanism of BiNPs. To accurately evaluate the therapeutic capabilities of BiNPs and uncover the microbial response to these elements, it is imperative to conduct in vivo studies. In the context of biological systems, in vivo, investigations are considered essential for providing a comprehensive understanding of their application. Therefore, it is imperative to conduct further investigations into the activity of BiNPs at the structural, genetic, and proteomic levels [138].

### Cobalt nanoparticles

Co oxide NPs are put to use in a wide variety of industries, from the medical sector to the production of sensors and magnetic materials to electrochemical systems and smart absorbers and catalysts. The colony formation of the gram-positive and gram-negative bacteria investigated was significantly reduced during the assessment of the antibacterial capabilities of the synthesized Co oxide NPs. An advantageous antibacterial activity against gram-negative bacteria is shown by a chemical contacting Co oxide NPs. As yet, no particular mechanism has been proposed to explain how the Co oxide NPs kill the bacteria. The synthesized Co oxide NPs have excellent antibacterial characteristics, making them suitable for application in the production of antibacterial dental and medical devices [139]. The objective of this study was to incorporate antibacterial properties into pits and fissure sealant (PFS) as a means to address the significant clinical issues associated with PFS, including microleakage and secondary caries. The pH-dependent incorporation of minocycline (MNC@CO) into cobalt oxide NPs was carried out by researchers, who subsequently characterized the resulting composite material. The purpose of this investigation was to assess the antibacterial efficacy of MNC@CO against *S. sobrinus*. The experimental groups with concentrations of 2.5% and 5.0% have demonstrated a statistically significant antimicrobial effect against *S. sobrinus* when compared to the control group (p < 0.05). The MNC@CO doped PFS with a concentration of 5.0% exhibited the greatest release of MNC at various pH levels, particularly at pH 5.0 and 3.5. The PFS incorporated with 2.5% MNC@CO exhibited the highest compressive strength of 110 MPa during a 70-day testing period, surpassing the compressive strengths of the PFS doped with 5.0% MNC@CO (75 MPa) and the control sample (80 MPa). The flexural strength of both experimental groups was lower for both time points (24 h and 30 days) than for control. In conclusion, the present study found that 2.5% MNC@CO doped PFS showed considerable anti-biofilm potential without compromising mechanical properties [140].

One of the most affordable transition metals is co, however, its antibacterial properties in its nano form have not been well investigated. It is also inadequate to compare CoNPs with bulk Co and conventional antibacterial. Investigations demonstrated that from 0.125 to 128.0 g/ml, Co NPs were shown to affect *S. aureus* and *E. coli*. *E. coli* was more successfully inhibited by the CoNPs zone of inhibition than *S. aureus*. Compared to bulk Co, oxytetracycline, and gentamicin, CoNPs performed noticeably better. At the majority of concentrations, the activity index and fold rise of CoNPs were greater. In conclusion, CoNPs showed superior antibacterial activity against *S. aureus* and *E. coli* compared to other investigated substances, especially at lower doses. Their usage may be expanded in many biomedical domains in the future. In conclusion, the CoNPs are superior to traditional antibacterials in their bulk form when it comes to fighting *S. aureus* and *E. coli*, especially at lower doses. Future uses of Co NPs' robust antibacterial properties might expand into other industries and bring about a significant transformation in the medical area [64]. A novel low-dimensional Co(II) coordination complex was synthesized using solvothermal reaction conditions by integrating an unsymmetrical tetracarboxylic acid based on a semi-rigid ether with the auxiliary dipyridyl ligand 4,4′-bipyridine (bipy). Both its therapeutic efficacy and the mechanism by which it works against chronic periodontitis were assessed. Real-time RT-PCR findings revealed that the chemical may dose-dependently reduce the relative expression levels of the survival genes expression in *P. gingivalis*. Inhibiting the expression of *P. gingivalis* survival genes, the chemical shows promise as a potential therapy for chronic periodontitis [141].

Because of their unique antioxidant, antibacterial, antifungal, anticancer, larvicidal, antileishmanial, anticholinergic, wound-healing, and antidiabetic capabilities, cobalt and cobalt oxide, NPs have a wide range of medicinal uses. To further complicate matters, cobalt, and cobalt oxide NPs have been synthesized using a variety of chemical and physical processes, some of which may be related to eco-toxicity, cost-effectiveness, high energy, and time consumption. The utilization of biotic resources including plant extract, microbes, algae, and other biomolecules like starch and gelatin has led to the development of a new approach that is safe for the environment, straightforward, simple, and quick. More benefits may be gained by using biogenic cobalt and cobalt oxide NPs than by using conventional physicochemical synthesis techniques [142]. However, there has been a dearth of research on the efficacy of CoNPs for periodontitis therapy. As a result, it is important to study CoNPs in depth to cure to cure periodontitis.

### Copper nanoparticles

Copper (Cu) NPs have a high level of biological activity, are very inexpensive, are environmentally benign, and have the potential to be effective multifunctional antibacterial agents. Other metals, ceramics, and polymers may easily mix and bond with CuNPs, and the products show physiochemical stability. CuNPs are thus among the metal NPs that are often employed in dentistry. Dental products including dental amalgam, restorative types of cement, adhesives, resins, endodontic-irrigation solutions, obturation materials, dental implants, and orthodontic archwires and brackets have all benefited from the usage of CuNPs to improve their physical and chemical qualities [143]. CuNPs have various metallic qualities connected to dental uses as well as antibacterial activity. These NP composites are simple to produce with currently available dental materials and are reportedly physiochemically stable. However, they only have very limited clinical use. CuNPs have mostly been investigated in dentistry as a modifier in amalgam and antibacterial agents. Recent studies on a wide range of dental materials have shown that CuNPs may be incorporated into several different settings. Research has shown that the carboxyl group of bacterial lipoproteins contains a negative charge, which attracts positive Cu ions. Cu ions interact with bacterial cell membranes, changing the membrane's permeability and enabling Cu ions to enter the cells. Cu ions modify cellular structures and proteins when they combine with phosphorus- and sulfur-containing macromolecules (such as DNA). The cell's biochemical functions are hampered and eventually die as a result of this modification. Enzyme activity is hampered, however, by Cu ions. They interfere with DNA or protein synthesis, render their enzymes inactive, and stimulate the generation of hydrogen peroxide. Furthermore, NPs interact with the sulfhydryl group of protein molecules, causing the molecules to become denatured. At the same time, the bacterial cell membrane becomes damaged, allowing DNA, ribonucleic acid, proteins, and cytoplasm to escape. In the evolution of dental material characteristics, CuNPs serve a dual function. Incorporating CuNPs into dental materials has the potential to boost their physio-mechanical characteristics and introduce or boost their antibacterial activity. This will provide light on the advantages and disadvantages of using CuNPs in dental practice, and open up a new avenue for dental biomaterials research [144].

The research set out to develop an antibiotic release system for periodontal treatment using chitosan and CuNPs, and then evaluate its efficacy against *A. actinomycetemcomitans* in a laboratory setting. A biocompatible system including CuNPs was synthesized out of chitosan, starch, and ascorbic acid. Solid sponges and gel spheres with a Cu concentration of 100 g/mL were made using CuNPs/chitosan gel nanocomposites. Nanometric Cu particles were proven to produce sponges and gel spheres out of chitosan. The development of *A. Actinomycetemcomitans* were stymied by these substances. The sphere nanocomposites were more stable in saliva and showed a prolonged release of Cu at amounts effective against bacteria. Nanocomposites containing CuNPs and chitosan show promise as a platform for developing targeted treatments for periodontitis [65].

Biologically synthesized AgNPs and CuNPs with the potential to display antibacterial action against several multidrug-resistant (MDR) microorganisms have been extracted from the fungus Shizophyllum commune. TEM, FTIR, dynamic light scattering, and ultraviolet–visible spectroscopy was used to determine the characterization. It was determined that the NPs were uniform in size and shape. Furthermore, the agar well diffusion technique was used to test the NPs' antibacterial activity against multidrug-resistant bacteria such as *Salmonella abony*, *Klebsiella pneumoniae*, *E. coli*, and *S. aureus*. These biosynthesized NPs have been shown to have a variety of biological uses, including the elimination of harmful bacteria and the bio-control of fungal strains [145]. Dental restorative materials are greatly impacted by pathogenicity due to improper adhesion and colonization by bacteria in the oral cavity. Therefore, scientists find that including tiny CuONPs into the sodium aluminosilicate ceramic material makes for an effective anti-*S. mutans* material investigation demonstrates that the composite ceramic is very biocompatible and effective against *S. mutans* (up to 99.99% antibacterial rate). The Cu ions in the composite ceramic prevent *S. mutans* from replicating its DNA, damage its respiratory system, and prevent its cell membrane from functioning properly, killing the bacterium. The antimicrobial properties of the composite ceramics correlated with the concentration of CuNPs present, and Cu ions were continuously released from the ceramics' interior to kill *S. mutans* within 10 days by altering membrane permeability, impeding respiratory chain activity, and interfering with gene replication [66] (Table 2).

**Table 2:** Effects of different NP on periodontitis

Nanoparticles	Characteristics	Effects on periodontitis	Study type	Refs.
AuNPs	45 nm AuNPs, anti-inflammatory effect, and improve periodontal inflammation	By directly modulating hPDLCs and indirectly modulating macrophage phenotypes, AuNPs could create a microenvironment with limited inflammatory cytokine levels and reparative cytokines like BMP-2, thereby inducing PDLC differentiation, periodontal tissue regeneration, and the prevention of periodontitis progression	In vivo and in vitro	[70]
AgNPs	1–100 nm, smaller particle size, higher surface area, and quantum confinement effects	Using AgNPs as an alternative to topical antiseptics and antimicrobial agents, as well as in combination with other antimicrobial agents for a synergistic impact and local drug delivery during periodontal treatment, provides a window of opportunity for further study in the field. CHX and AgNPs are both potent antimicrobials that are effective against a wide range of periodontal and oral infections	In vitro	[55]
ZnONPs	Antimicrobial properties, the absorption peak of ZnNPs was in the range of 230–330 nm	Individuals with chronic periodontitis have higher levels of ALT enzyme activity in their saliva compared to healthy controls, and ZnONPs have been shown to increase ALT enzyme activity in these individuals	20 patients with chronic periodontitis disease	[98]
MNPs	Simple synthesis, intrinsic antimicrobial activity, low toxicity, and high versatility	In vitro antibacterial investigations showed that antibacterial MNPs could eliminate bacterial biofilms using magnetic inductivity and the nanosize effect of NPs in conjunction with the antibacterial impact of PHMB, achieving a clearance rate of close to 80%	In vivo and in vitro	[118]
NiNPs	Cheap enough for intensive use, antibacterial activities, and safe, NPs obtained are smaller than 25 nm with low polydispersity	All *S. epidermidis* clinical isolates were shown to be capable of biofilm generation in the investigation. The production of biofilm was shown to be suppressed by NiNPs. Factors that contribute to the progression of these infections include the capacity to produce biofilms, PIA, biofilm-associated protein, PGA, SEIL and SEC3, PSMs, Clpxp, and extracellular matrix-binding protein	In vitro	[124]
BiNPs	Bactericidal, fungicidal, antiparasitic, 4–22 nm, and antibiofilm agents	It was shown that the main size of BSS-nano is between 4 and 22 nm and has a polygonal form. Antimicrobial BSS-nano may be used in dental fillings and antiseptics	In vitro	[135]
CoNPs	Antibacterial properties, Cobalt (Co) is one of the cheaper transition metals	CoNPs have not been compared to bulk Co or to other common antimicrobials. Investigation showed that between 0.125 and 128.0 g/ml, CoNPs were effective against *S. aureus* and *E. coli*. CoNPs showed a larger zone of inhibition against *E. coli* than they did against *S. aureus*. When compared to bulk Co, oxytetracycline, and gentamicin, CoNPs were superior	In vitro	[64]
CuNPs	High biological activity, comparatively low cost, ecological safety, and antibacterial agents	Solid sponges and gel spheres containing 100 g/mL of copper were made using CuNPs /chitosan gel nanocomposites. The development of *A. Actinomycetemcomitans* was stymied by these substances. Nanocomposites containing CuNPs and chitosan show promise as a platform upon which to build site-specific treatments for periodontitis	In vitro	[65]
TiO_2_NPs	Antibacterial, anti-inflammatory, biocompatibility	In PDL cells, TiO2NPs increased COX-2 mRNA and protein expression. After being exposed to TiO2NPs, ERK1/2, and Akt were rapidly activated, perhaps upstream of NF-κB. Following treatment with TiO2NPs, intracellular ROS production increased in PDL cells	In vitro	[111]

## Advantages and the perspective of comparison of metal nanoparticles for the development of therapeutic methods

Traditional antibacterial medications are ineffective against the biofilm that accumulates on human wounds, but multiple studies have demonstrated that nanomaterials may help fight bacterial drug resistance. Among the various chemotherapeutic medications, metal ions including Au, Ag, Zn, and Cu have been studied extensively for their effectiveness against periodontal and peri-implant bacteria [146]. Benefits of using NPs as antibacterial agents include their effectiveness against antibiotic-resistant bacteria, their ability to target diverse microbes, and their use as effective antibiotic transporters. The potential applications of NPs in the treatment of periodontal diseases are vast. Antimicrobial research has shown that NPs may break apart bacterial biofilms and spread them. Biofilm-encased periodontal bacteria are likely to be eliminated by NPs. However, clinical investigations are needed to confirm whether or not NPs can disperse biofilms produced by periodontal bacteria [147]. They work well against the gram-negative and gram-positive bacteria that cause periodontitis and peri-implantitis, including *Prevotella intermedia*, *F. nucleatum*, and *P. gingivalis*. The large surface area of the biosynthesized AgNPs increased interactions between flavonoids and the cell surface or bacteria, hence amplifying the flavonoids' physiological effects. By boosting contacts between flavonoids and the cell surface or bacteria, the biosynthesized AgNPs improved the flavonoids' biological effects [86, 148]. There is a direct correlation between AgNP size and their bactericidal action, stability, and biocompatibility. The optimal size for AgNPs is between 10 and 15 nm, with activity peaking at 15 nm. Since smaller AgNPs have a greater surface area to NP volume ratio, they are better able to interact with cell membranes. In addition, *S. mutans* growth, adhesion, and biofilm generation have all been shown to be inhibited by AgNPs, as shown in in vitro investigations [149]. Using methods that remove the plant material may allow for the cheap, sustainable, and ecologically responsible production of AgNPs. Plants, because of their phytochemical components, offer a superior, non-toxic, and more cost-effective alternative to both physical and chemical methods. Since the effectiveness and toxicity of AgNP rely on both their size and shape, synthesis methods and procedures have lately been the focus of a considerable amount of scientific interest. AgNPs may be made by physical, chemical, or biological means. Physical techniques need a lot of energy to maintain the high pressure and temperature necessary for the reaction, while the chemical method is dangerous and costly. The disadvantages of nanomaterials have inspired the development of biochemical techniques or biosynthesis, such as the use of biomolecular extracts from plants, to overcome issues including toxicity towards bone cells, varying biocompatibility depending on size, surface, and composition, and high cost [150]. Because of their adaptability and versatility, AuNP may be altered to improve their stability, biocompatibility, and antibacterial characteristics. The antibacterial properties of loaded antibacterial medicines may be enhanced by using AuNPs as drug carriers. Meanwhile, the use of AuNPs in oral biology is on the rise. Dental disease is only one area that might benefit from AuNPs' unique qualities, such as their adaptability in size, shape, surface properties, optical properties, biocompatibility, low cytotoxicity, high stability, and multi-functional potential. Because of their ability to inhibit the growth of fungi and bacteria, AuNPs may be introduced into certain biological materials to increase their use by providing such materials with antibacterial capabilities [7].

**Fig. 6:**
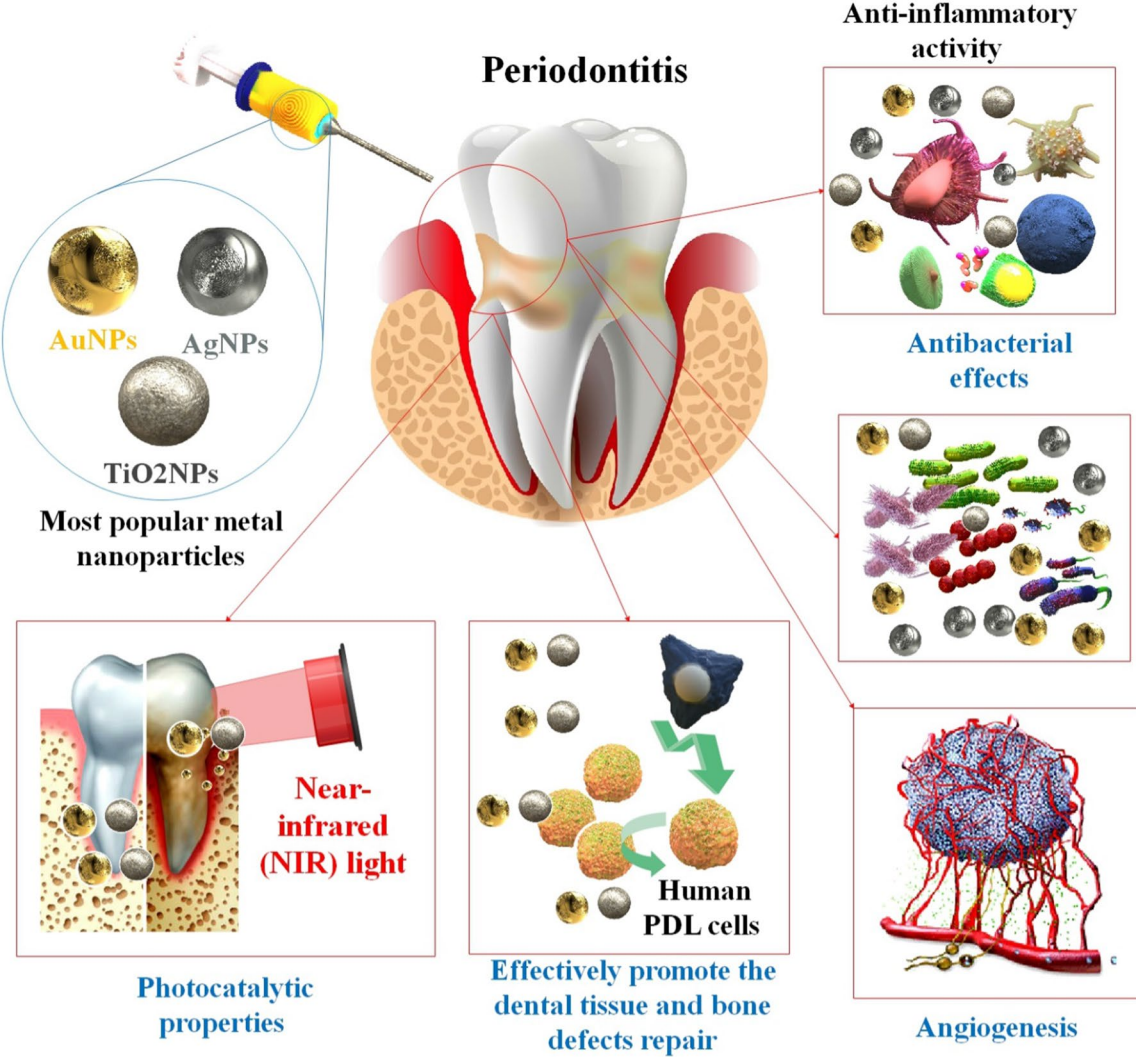
Various effect of the most popular metal NPs as a therapeutic approach for periodontitis

When compared to AgNPs, the most commonly commercially utilized ZnONPs offer several benefits, including a cheaper price and a whiter look. Toxic heavy metals react with proteins, binding their molecules. Heavier metals have a powerful interaction with the thiol groups of essential enzymes, rendering them useless. Proteins are thought to be deactivated and denatured once metal NPs like Ag attach to their functional groups. Researchers revealed that exposure to surface-functionalized TiO2NPs and UV light did not affect ALP activity in intact heterotrophic biofilms. However, at concentrations of ZnONPs far lower than those seen in complete biofilms, the ALP enzyme that is secreted by *E. coli* is severely inhibited [151, 152]. Indeed, in in vivo toxicity experiments, both ZnONPs and TiO2NPs have shown considerable antibacterial efficacy against oral bacteria at non-toxic doses. This means that ZnO and TiO2NPs might potentially be used as nano-antibiotics in the creation of various oral hygiene products including mouthwashes and dental pastes. Dental biofilms and oral planktonic bacteria may be managed with fewer adverse effects and less antibiotic resistance if these NPs are used as an alternative [153]. When compared to their metallic, semiconducting, silica- or carbon-based counterparts designed for pharmacological and biological applications, MNPs offer additional desirable features. By applying a magnetic field externally, magnetic NPs might be remotely directed to specific locations. Furthermore, the application of a varying magnetic field causes MNPs to waste energy as heat, leading to a localized rise in temperature. All of these methods seem to be successful, but MNPs are among the most promising concerning clinical translation in the area of antibiotic treatment. MNPs are designed for pharmaceutical and biological applications due to their unusual physical features and their ability to work at the cellular and molecular levels. Furthermore, MNP drug delivery methods enhance localization, reducing the dosage of medications required to achieve the desired effect. These benefits result from the antibacterial qualities present in the bulk form of some metals, such as Zn, Ag, and Cu [154]. Many advances have been made, but the fight against tooth disease is far from over. To create MNPs with sufficient biocompatibility, new synthesis processes, and methodologies are still required. MNPs-based composites with fine microstructures and superior biomedical capabilities need more efficient ways of preparation [155]. A summary of the performance of various types of metal NPs in the treatment of periodontitis is shown in Fig. 6.

Cu's antibacterial qualities and low toxicity make it a desirable research material in the fields of medicine and dentistry. Copper's metal ions or the oxidized Cu ions produced by CuNPs (1–100 nm) are responsible for the antibacterial actions. Moreover, since Cu may be produced either naturally or via chemical synthesis, CuNPs are processed at a low cost. In addition, CuO NPs may be readily formed by oxidation in air or water. Cu particles, like other metal NPs used in dentistry, have a wide range of nano-sizes and shapes, a one-of-a-kind distribution, and a high surface-to-volume ratio. These characteristics improve the NPs’ antibacterial efficacy, biocompatibility, and bio-physiochemical functionalization. CuONPs have been demonstrated to be antibacterial and to prevent the production of biofilms. The antimicrobial activity of Cu NPs is improved by their high surface area/volume ratio. Despite extensive research, the precise method by which CuNPs kill microorganisms remains unclear. The bactericidal activity of CuNPs against *E. coli*, *B. subtilis*, and *S. aureus* is superior to that of AgNPs, another kind of NP often employed in biomedical research. Antimicrobial and other metallic characteristics of interest for dental applications are present in CuNPs. It is simple to create these NPs composites using standard dental materials, and they are reported to be physiochemically stable. However, their use in the clinic is quite restricted. As a modifier in amalgam and an antibacterial agent, CuNPs have received the majority of the research attention in the dental field. Dental cement, restorative materials, adhesives, resins, irrigating solutions, obturations, orthodontic archwires and brackets, implant surface coatings, and the bone regeneration process are all reported to benefit from the addition of CuNPs, the subject of recent research. CuONPs are less expensive, chemically stable, and safe for the environment than AgNPs [144].

However, their use in the clinic is quite restricted. As a modifier in amalgam and an antibacterial agent, CuNPs have received the majority of the research attention in the dental field. Dental cement, restorative materials, adhesives, resins, irrigating solutions, obturations, orthodontic archwires and brackets, implant surface coatings, and the bone regeneration process are all reported to benefit from the addition of CuNPs, the subject of recent research. In comparison to AgNPs, CuONPs are more environmentally friendly, less costly, and more stable chemically. Cu and NiNPs have been found to exhibit bactericidal action in laboratory studies. They have not been synthesized in aqueous solution, however, since their characteristics may be compromised by the addition of stabilizers such as polymers, ligands, salts, etc. Furthermore, Cu-Ni bimetallic NPs' antibacterial activities have not been investigated till recently. To synthesize the desired NPs, several variables, including pH and reducing agent concentration, are used. Despite claims that nickel oxide nanotubes may be synthesized with potent antibacterial properties, the present study demonstrated that NiNPs have neither bactericidal nor bacteriostatic activity. Finally, the antibacterial action of bimetallic Cu-Ni NPs has not been reported before. Nickel’s bacteriostatic activity and the fact that bimetallic Cu-Ni particles are bigger than CuNPs and NiNPs may both be to blame for this behavior. When creating NPs, the experimental settings used are crucial. To create stoichiometric metal nanoalloy (bimetallic) NPs, pH plays a crucial role. The redox potential of the metals is what decides this. Successfully characterizing the NPs. Since the sample is exposed to the atmosphere during characterization, oxides develop, making the study of zero-valent metal compounds challenging. The produced NPs have a polydispersity of less than 5% and a size of less than 25 nm [127]. The research and refinement of metal NP production technologies for use in periodontitis therapy are crucial. These NPs are useful in the treatment of this condition thanks to their antibacterial and anti-inflammatory capabilities, as well as their simple and inexpensive manufacturing procedure.

Metal NPs are being employed for periodontitis therapy and other dental applications due to their unique properties; nevertheless, these NPs should be thoroughly investigated for any harmful side effects to guarantee their safe usage. Furthermore, the cost of raw materials and processing, their availability, their sustainability towards end-use, and their recyclability are all factors that must be addressed when using the circular economy idea [156, 157]. When it comes to treating periodontitis, the major antibacterial mechanism underlying the activity of various metal NPs, such BiNPs, and CoNPs, is still not fully known. In vivo, studies are crucial for accurately gauging the therapeutic potential of metal NPs and uncovering the microbial response to these variables. In-vivo studies are crucial for providing a complete explanation of their function in living systems [116]. In addition, the journals discussed here contain evidence that suggests research into the use of metal NPs to treat periodontitis is still in its early stages. A large proportion of the studies reviewed here are technically focused investigations that often lack a principal expense/advantage analysis and specifications of the primary steps of each investigation. The mechanisms of metal NPs’ cellular absorption and their antibacterial effects in periodontitis therapy need further investigation. It's worth noting that most mechanisms for site-specific transfer perform well in vitro but poorly in vivo testing. Therefore, in vivo, testing for periodontitis therapy might benefit this study.

## Conclusion

Several traditional treatments for periodontitis exist, each targeting different aspects of the disease's etiology and pathophysiology. Antibiotic and antimicrobial drug treatment is widely used. However, traditional approaches are rendered useless due to medication resistance and the emergence of unwanted effects. Metal NPs are the most promising antibacterial material because of their high specific surface area to volume ratio, prolonged shelf life, and biocompatibility. Researchers' interest in metal NPs growing as a result of the creation of strains that are resistant to drugs and the improvement of microbial resistance to antibiotics. Due to the NPs' tiny size, they may enter the biofilm matrix and make direct contact with the bacterial cells, which inhibits the biofilm. As we move closer to practical applications, it's expected that antibacterial research will improve further. Metal NPs have efficient antibacterial capabilities. Given the recent advancements and ongoing efforts in improving particle synthesis efficiency and examining their biomedical applications, it is hoped that the implementation of our strategy on a large scale and their commercial applications in medicine and health care will be very helpful in the upcoming years. It is anticipated that antimicrobial research would advance more as we get closer to real-world applications. The antibacterial properties of metal NPs are very effective. With recent and ongoing improvements to particle synthesis efficiency and research into their biomedical applications, it is hoped that widespread implementation of investigation strategy and their commercial applications in medicine and health care will prove immensely beneficial in the years to come.

## Data Availability

Not applicable.
